# *Pulsatilla chinensis* functions as a novel antihyperlipidemic agent by upregulating LDLR in an ERK-dependent manner

**DOI:** 10.1186/s13020-024-01044-3

**Published:** 2024-12-19

**Authors:** Wei-fang Song, Rui-jun Wang, Rui-xin Yao, Qiu-yan Jiang, Juan Feng, Kun Luo, Zheng-han Di, Cheng-mei Ma, Lan Xie

**Affiliations:** 1 Department of Pathophysiology, Fenyang College, Shanxi Medical University, Fenyang, 032200 China; 2 Medical Systems Biology Research Center, Tsinghua University School of Medicine, Beijing, 100084 China; 3 National Engineering Research Center for Beijing Biochip Technology, Beijing, 102206 China; 4 College of Health Science and Environmental Engineering, Shenzhen Technology University, Shenzhen, 518118 China

**Keywords:** High-throughput screening, *Pulsatilla chinensis*, RNA-seq, LDLR, ERK, Pulsatilla saponin D

## Abstract

**Background:**

*Pulsatilla chinensis* (PC) is a traditional Chinese medicine (TCM) known for its beneficial activities. It has been historically used to treat dysentery, vaginal trichomoniasis, bacterial infections, and malignant tumors. The therapeutic potential of PC in the management of hypercholesterolemia remains largely unexplored.

**Methods:**

A high-throughput screening based on high-throughput sequencing was conducted in HepG2 cells to construct gene expression profiles for several hundred TCMs. In vivo evaluation of the efficacy of PC was performed using rats with hypercholesterolemia. Transcriptome analysis was carried out on PC-treated rat livers and HepG2 cells to investigate the mechanism of action of PC in vitro. The findings were further validated using RT-qPCR and western blot techniques.

**Results:**

PC was identified as similar to Rhizoma Coptidis based on signature genes related to metabolism. Administration of PC via gavage in rats with hypercholesterolemia for 11 weeks resulted in substantially reduced serum total cholesterol and low-density lipoprotein (LDL) cholesterol and ameliorated fatty liver. Transcriptome analysis revealed that PC regulated various pathways associated with lipid metabolism. The LDL receptor (LDLR), a key player in cholesterol metabolism, was upregulated by PC both in vivo and in vitro. It was discovered that PC achieved this upregulation by activating extracellular regulated protein kinase (ERK) signaling in HepG2 cells. To uncover the major bioactive components responsible for the anti- hypercholesterolemia effect of PC, two major saponins, named Pulsatilla saponin D (PCD) and PC anemoside B4 (PCB4), were assessed. PCD, but not PCB4, was identified as the active ingredient responsible for the upregulation of LDLR by PC.

**Conclusion:**

These findings demonstrated that PC acts as an antihypercholesterolemic agent by upregulating LDLR in an ERK-dependent manner and holds potential in the treatment of hypercholesterolemia.

**Supplementary Information:**

The online version contains supplementary material available at 10.1186/s13020-024-01044-3.

## Introduction

Cardiovascular diseases are the leading cause of both death and disability worldwide, and hypercholesterolemia is a known risk factor for the progression of cardiovascular diseases [27, 31]. Hypercholesterolemia refers to elevated cholesterol, mainly low-density lipoprotein cholesterol (LDL-c) levels in serum. The prevalence of hyperlipidemia is estimated as 36.1% in US adults aged 20–44 years in 2017–2020 [1]. Statins are the first-line antihyperlipidemic therapeutics which function through inhibition of 3-hydroxy-3-methyl-glutaryl-coenzyme A (HMG-CoA) reductase. However, the adverse effects (including muscle myopathy and hepatic function disruption) caused by statins [24] urgently require the discovery of therapeutic agents both effective and safe.

Traditional Chinese medicine (TCM) has demonstrated curative effects on hypercholesterolemia [9]. TCM contains a variety of components, which potentially act on multiple molecular targets to achieve a stronger efficacy. In the meanwhile, TCM is relatively safer and has fewer side effects than western medicine [37]. Interestingly, most of the antihypercholesterolemic TCMs are bitter tasting herbs, as recorded in *Chinese materia medica* [22]. For instance, *Coptis chinensis* is a typical bitter tasting herb, and the serum total cholesterol (TC) and serum LDL-c levels were significantly reduced by *C. chinensis* extract in rats [35]. In particular, berberine, the most abundant alkaloid in *C. chinensis*, has shown cholesterol-lowering effects in animals [13, 32]. A clinical trial showed that berberine could effectively treat type 2 diabetes and dyslipidemia [42]. Berberine functions through a distinct mechanism from statins in that it upregulates low-density lipoprotein receptor (LDLR) expression at the post-transcriptional level. This molecule stabilizes the 3′ untranslated region (UTR) of LDLR and is independent of sterol-regulatory element binding proteins (SREBPs) but dependent on extracellular regulated protein kinase (ERK) activation [12]. In addition, berberine promotes hepatic LDLR expression at the transcriptional level by activating the N-terminal kinase (JNK)/c-jun pathway [17].

Gene expression signatures of cells can reflect cellular perturbation in response to drug treatment. RNA annealing, selection, and ligation with next-generation sequencing (RASL-seq) is a method to achieve parallel and quantitative analysis of gene transcription profiles under thousands of different conditions [25]. RASL-seq has been successfully applied in drug discovery and has helped exploring the effects of different TCM formulae against SARS-CoV2 [4, 29]. TCM is a precious resource for drug discovery, and we aimed to screen TCMs with efficacy against hypercholesterolemia using RASL-seq. We used the liver-derived cancer cell line HepG2 to study the gene expression profiles after treatment with hundreds of bitter-tasting TCMs. *C. chinensis* and berberine were included as positive controls. A gene set of 639 unique genes related to glucose and lipid metabolism was designated signature genes.

The rhizoma of *Pulsatilla chinensis* (PC) is a medicinal herb recorded in the Chinese Pharmacopeia (2020) that has been widely used for adjunctive treatment of dysentery, vaginal trichomoniasis, bacterial infections and malignant tumors [18, 43]. Chemical and pharmacological investigations indicated that the major bioactive constituents of PC are triterpenoid saponins [36]. Recent studies have demonstrated that Pulsatilla saponin D isolated from PC (PCD) and anemoside B4 in PC (PCB4) are effective saponins [8, 41]. PCB4, a marker for quality control of the herb, as documented in the Chinese Pharmacopoeia, showed the highest content in the herb and has antitumor, anti-inflammatory, antiviral and immunomodulatory activities [7]. Recent studies also showed that PCD exhibits strong activity against liver tumors in vitro and in vivo [38].

In the present study, we screened PC as a metabolic regulatory agent based on RASL-seq. Then, we evaluated the efficacy and mechanism of the antihyperlipidemic activity of PC in vitro and in vivo. Transcriptome analysis of the PC-treated rat livers and HepG2 cells revealed that PC regulated a series of lipid metabolism-related pathways. PC substantially increased the expression of LDLR through activation of ERK, thus increasing the LDL-c absorption capacity of cells. PCD but not PCB4 functions as the principle bioactive component of PC mediating the upregulation of LDLR. This study represents an investigation into the cholesterol-lowering properties of PC, unveiling a novel application for this traditional medicine, thus exemplifying the concept of “a new use of old medicine”.

## Materials and methods

### Materials

TCMs were purchased from Anguo City Traditional Chinese Medicine Market (the complete list is summarized in Supplementary Table 1). Berberine was obtained from the National Institutes for Food and Drug Control (Beijing, China). U0126 was obtained from Cell Signaling Technology (CST, Boston, MA). PCD and PCB4 were purchased from Stronger Technology Co., Ltd. (Beijing, China). Primary antibodies for GAPDH and ERK/p-ERK were obtained from CST, and an antibody for LDLR was obtained from Abcam (Cambridge, UK). All secondary antibodies were purchased from CST.

### TCM herb extraction

Each TCM was weighed and ground into powder. Then, the powder (100 g) was extracted with 90% ethanol for 3 h in a B-811 Soxhlet extractor (BUCHI Labortechnik AG, Flawil, Switzerland) following the manufacturer’s instructions. After extraction, the resultant ethanol extracts were concentrated and dried under a vacuum to a constant weight. The extracts were stored at − 20 °C for further use.

### RASL-seq

First, HepG2 cells were distributed in 384-well plates and treated with different TCM extracts for 24 h. Then, RASL-seq was performed as previously described [16]. Briefly, cell samples were lysed, and all the designed probes were added to cell lysates. The samples were transferred to the Agilent Bravo automated liquid handling platform and the Agilent bench robot (Agilent Technologies, Santa Clara, CA). RNA annealing, selection and ligation were automatically performed. The ligated pair probes were eluted and amplified by PCR [30]. The PCR products were purified and quantified as the library for high-throughput sequencing using an Illumina HiSeq X Ten sequencer (Illumina, San Diego, CA).

### Animal studies

Male 6- to 8-week-old Sprague–Dawley rats (180–220 g) were obtained and maintained at the Beijing Vital River Laboratory Animal Technology Co., Ltd. (Beijing, China). All procedures were approved by the Institutional Animal Care and Use Committee of Beijing Vital River Laboratory Animal Technology Co., Ltd. After 1 week of acclimation, the animals were separated into two groups. One group was given a normal diet (ND) group (n = 8), and the other group (n = 16) was given a high-cholesterol and high-fat diet (HCHFD) [6]. The HCHFD was prepared by adding 1.2% cholesterol and 15% lard to the normal chow diet. After four weeks, the group given the HCHFD was randomly divided into two subgroups (n = 8 per group). One group was the model group fed the HCHFD plus distilled water (HCHFD group). The other group was fed the HCHFD plus PC (200 mg/kg/day) (HCHFD + PC group). For the HCHFD + PC group, PC was dissolved in distilled water and administered via gavage every day, while an equal volume of distilled water was used in the ND and HCHFD groups. Blood samples were collected, and biochemical indexes and serum lipid profiles were measured using kits from Waka Pure Chemical Industries (Kyoto, Japan) at 0, 4, 8 and 11 weeks. At the end of the 11-week period, all rats were sacrificed after 16 h of fasting. Blood samples were collected, and serum was isolated after centrifugation (1200*g*, 10 min, 4 °C). Livers were removed, rinsed in ice-cold PBS, and prepared for subsequent analysis.

### Hematoxylin–eosin (H&E) staining and Oil red O staining

Liver tissues were collected, fixed in 4% formaldehyde for 24 h at 4 °C, dehydrated through an ethanol series (50–100%), cleared in xylene and embedded in paraffin. The tissue was cut into 5 µm thick sections, dewaxed in xylene, stained with hematoxylin, rehydrated through a 70–100% ethanol series, and further stained with eosin. Finally, the tissue sections were cleared in xylene and sealed with neutral balsam.

For Oil red O staining, frozen sections were prepared (6 µm) from liver tissues and fixed in 50% ethanol. Subsequently, the sections were stained with Oil red O for 8 min and split with 50% ethanol, washed with tap water, and counterstained with hematoxylin. Finally, the sections were sealed with neutral balsam. The sections were photographed using a light microscope linked to a digital CCD camera (Nikon, Tokyo, Japan).

### Cell culture

The human hepatocellular carcinoma cell line HepG2 and the human normal hepatocyte cell line HL-7702 were purchased from the Institute of Basic Medical Sciences, Chinese Academy of Medical Science (Beijing, China). HepG2 cells were cultured in DMEM supplemented with 10% fetal bovine serum and 1% penicillin–streptomycin solution (complete medium). HL-7702 cells were cultured in RPMI-1640 supplemented with 10% fetal bovine serum and 1% penicillin–streptomycin solution.

### Hyperlipidemia model establishment and PC treatment in vitro

HepG2 cells were plated in 12-well plates (2 × 10^5^ cells/well). The next day, the medium was discarded and cells were supplemented with complete medium containing oleic acid (0.2 mM) or oleic acid (0.2 mM) plus PC (125 μg/mL). After treatment for 24 h, total RNA was collected for cDNA synthesis and LDLR expression evaluation.

For the investigation of LDLR expression in a high cholesterol environment, HepG2 cells were treated with cholesterol (50 μg/mL) and 25-Hydroxycholesterol (4 μg/mL) for 6 h, followed by PC (125 μg/mL) or PC (125 μg/mL) plus U0126 (2.5 μM) treatment for another 24 h.

### CCK8 assay

Cell viability was assessed using a Cell Counting Kit-8 (Dojindo Molecular Technologies, Kumamon, Japan). Briefly, cells were plated in 96-well plates (2 × 10^4^ cells/well) at 37 ℃ with 5% CO_2_ for 24 h, and then treated with drugs at different concentrations for 24 h. The absorbance was recorded at 450 nm using a SpectroMax Spectrophotometer (SpectroMax Solutions Ltd., London, UK).

### RNA-seq and data analysis

TRIzol reagent (Invitrogen, Carlsbad, CA) was used to prepare total RNA samples from liver tissues or HepG2 cells. A cDNA library was constructed and subjected to sequencing by an Illumina Solexa sequencer. Differentially expressed genes (DEGs) were defined with a two-fold increase/decrease in expression with a *p*-value < 0.05.

For determination of the functional distribution of the potential targets of PC, KEGG pathway enrichment analysis was performed using the Database for Annotation, Visualization and Integrated Discovery (DAVID, http://david.abcc.ncifcrf.gov/). The enrichment of KEGG is represented by a *p*-value (*p* < 0.05 was considered significant).

### RNA isolation, cDNA synthesis and quantitative real-time PCR (qRT-PCR)

Total RNA was isolated from liver tissues or cells using TRIzol reagent according to the manufacturer’s instructions. cDNA was synthesized using 1 μg total RNA, 1 μL reverse transcriptase, and 10 μL of RT Buffer (Thermo Fisher Scientific, Rochester, NY) to a final volume of 20 μL. qRT-PCR reactions consisted of 3 μL of cDNA template, 1.8 μM of each primer, and 1 × SYBR Green Master Mix (Kapa Biosystems, Woburn, MA) in a final volume of 10 μL. The gene expression was normalized to that of GAPDH. Gene expression was quantified using a comparative method (2^−ΔΔCT^) [21].

### Protein extraction and Western blot

Total protein was isolated from cells or tissue samples using RIPA lysis buffer (Applygen Technology Inc., Beijing, China). The protein concentration was measured using the BCA method. Equal amounts of total protein were separated by SDS-PAGE electrophoresis, transferred to PVDF membranes, and blocked with 5% skim milk powder at room temperature for 1 h. Next, the PVDF membranes (Merck Millipore, Billerica, MA) were incubated with primary antibodies against target proteins at 4 °C overnight, followed by washing with TBST three times. The PVDF membranes were incubated with appropriate secondary antibodies at room temperature for 1 h. Protein bands were visualized using enhanced chemiluminescence reagents and analyzed with a gel documentation system (Bio-Rad, Hercules, CA).

### LDL uptake measurement

HepG2 cells were treated with or without PC for 24 h, followed by incubation with BODIPYTM FL-labeled LDL (Invitrogen) for another 2 h. Cells were rinsed three times with wash buffer at room temperature. Flow cytometric analysis was performed using the Calibur Flow Cytometer (BD, Franklin Lakes, NJ) [26]. The intracellular fluorescence intensity indicates the amount of BODIPYTM FL-labeled LDL absorbed by cells.

### Statistical analysis

Pearson’s product-moment correlation test was used for correlation analysis. Two-tailed unpaired Student’s t-test was used for statistical analysis between animal groups (HCHFD *vs.* ND; HCHFD + PC *vs.* HCHFD). Two-tailed paired Student’s t-test or one-way ANOVA was used for cellular analysis as indicated. Data are expressed as the mean ± standard deviation. A *p*-value < 0.05 was considered statistically significant.

## Results and discussion

### RASL-seq revealed PC as a metabolic regulatory agent

A total of 81 TCM crude extracts were prepared (Table S1) as candidates for RASL-seq; these TCMs were all bitter-tasting herbs, either bitter cold or bitter warm. DMSO was used as the solvent control. Rhizoma Coptidis and its main component berberine were included as positive controls. Three cultured varieties of Rhizoma Coptidis were included: *Coptis chinensis* Franch*.* (Weilian in Chinese), *Coptis deltoidea* C.Y. Cheng et Hsiao (Yalian in Chinese), and *Coptis teeta* wall. (Yunlian in Chinese). In addition, *Coptis chinensis* Franch. samples from five different growing regions were included: Dayi and Hongya from Sichuan Province, Lichuan from Hubei Province, and Shizhu from Chongqing municipality and Shanghai. Berberine was used at three concentrations: 10 μM, 50 μM and 100 μM.

The signature gene set was selected from 12 glucose- and lipid-related signaling pathways from the Kyoto Encyclopedia of Genes and Genome (KEGG) database, such as the human obesity signaling pathway and human fatty liver signaling pathway. A total of 639 probe pairs were designed to detect the expression of each unique gene based on RASL-seq (the pathway and its component genes are listed in Table S2). HepG2 cells were treated with TCM extracts at a concentration of 125 μg/mL (except berberine) or solvent control and were subjected to RASL-seq.

**Fig. 1:**
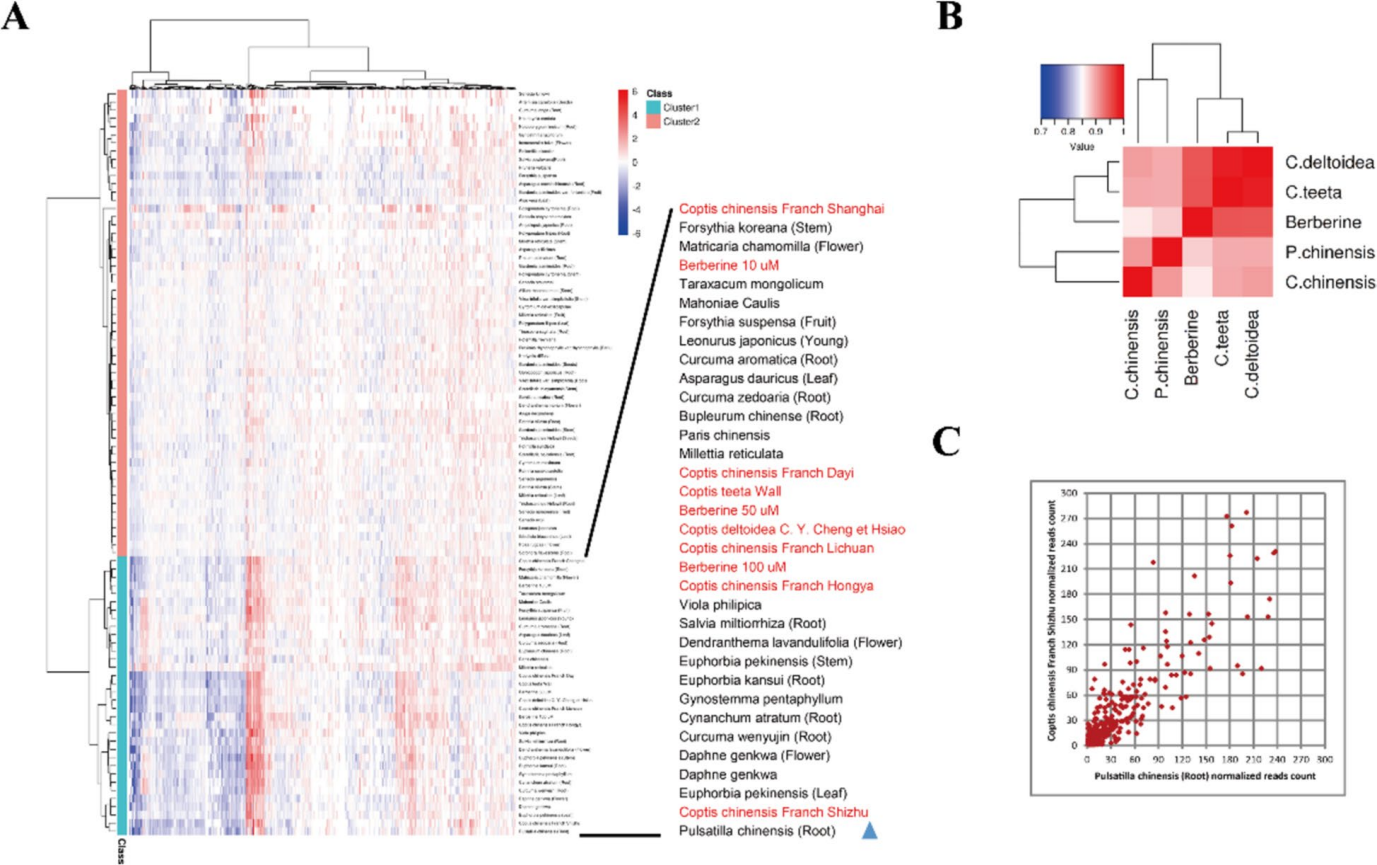
High-throughput screening revealed PC as a metabolic regulatory agent. **A** Heat map of the gene expression profiles of 81 TCMs, together with Rhizoma Coptidis and berberine. Red represents the upregulated genes; blue shows the downregulated genes. **B** Correlation coefficient matrix of PC, *C. chinensis, C. deltoidea* and *C. teeta*, the color in the heat map represents the correlation coefficient between each two groups of samples, and the color ranges from white (low correlation coefficient) to red (high correlation coefficient). **C** Scatter plots between PC and *C. chinensis* from Shizhu; *R*^*2*^ = 0.71. Each spot represents a differentially expressed gene

As shown in the heatmap in Fig. 1a, all the TCMs were roughly divided into two categories: cluster 1 and cluster 2. Cluster 1 included various *C. chinensis* samples and berberine at different concentrations. Interestingly, we found that PC was in this cluster and very close to *C. chinensis* Franch. from Shizhu. To further explore the similarity among PC, *C. chinensis* and berberine, we calculated the Pearson correlation coefficients of PC, *C. chinensis* and berberine according to the expression of genes in each sample. Notably, PC was similar to *C. chinensis* with a Pearson’s correlation coefficient of 0.91 (Fig. 1b). The scatter plot of the DEGs of PC and *C. chinensis* indicated that the two samples were very close, with an R^2^ of 0.72 (Fig. 1c). These results suggested that PC was similar to *C. chinensis*, based on metabolism-related gene signatures, and that PC has potential as a metabolic regulatory agent. Therefore, we aimed to explore the function and underlying mechanisms of PC in vivo and in vitro.

### PC ameliorated hypercholesterolemia and liver steatosis in rats

**Fig. 2:**
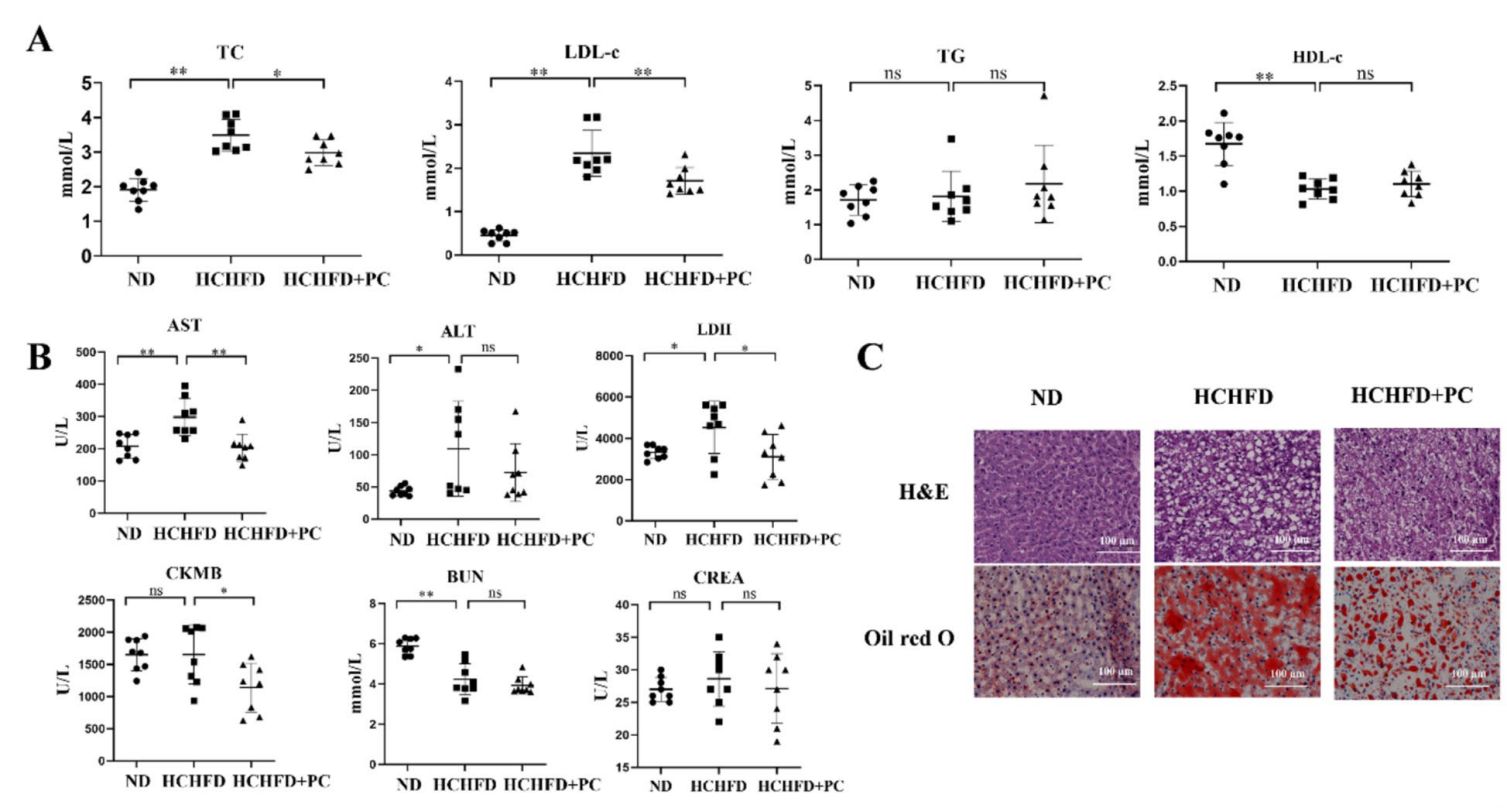
PC ameliorated hypercholesterolemia and liver steatosis in rats. **A** Serum levels of TC, LDL-c, TGs, and high-density lipoprotein cholesterol (HDL-c) in the rats of the normal diet group (n = 8), the HCHFD group (n = 8), and the HCHFD + PC group (n = 8). **B** Serum AST, ALT, LDH, CK-MB, BUN and CREA levels in the rats of the three groups. C Representative pictures of H&E staining and Oil Red O staining of the rats in the three groups. Scale bar = 100 μm. *p < 0.05; **p < 0.01; ns: not significant

To study the function of PC in vivo, we established an animal model of HCHFD-induced hypercholesterolemia to assess the effects of PC on the serum lipid levels. The TC and LDL-c of rats in the HCHFD model group were highly induced compared to those in the ND group (Fig. 2a) [6]. We used 200 mg/kg/day PC for treatment of rats; this concentration was equivalent to 31.7 mg/kg for humans (within the recommendation dose, *i.e.* 9–15 g in the Chinese Pharmacopeia). Intervention with PC for 11 weeks significantly lowered the serum levels of TC and LDL-c by 14.6% and 27.2%, respectively (Fig. 2a, HCHFD + PC *vs.* HCHFD, *p* < 0.05). The serum total triglycerides (TGs) and HDL-c were not altered by PC (Fig. 2a). The reduction magnitude of TC and LDL-c by PC is comparable with other animal studies for the evaluation of antihyperlipidemic effect of TCMs [14, 23, 39]. More importantly, similar results were observed in the clinical trials of hypolipidemic TCMs or natural products. For example, serum TC and LDL-c levels are lowered by 21.4% and 27.3% with red yeast rice treatment [2], 3.8% and 4.2% with rhubarb treatment [20], and 29% and 25% with berberine treatment [12], respectively, indicating that moderate changes in lipid levels have clinical significance.

PC was classified as a toxic TCM in the Chinese Pharmacopeia. Therefore, we evaluated the toxicity of PC by examining the serum levels of aspartate aminotransferase (AST), alanine aminotransferase (ALT), creatine kinase isozyme (CK-MB), lactate dehydrogenase (LDH), blood urea nitrogen (BUN) and creatinine (CREA) after PC administration. The levels of AST, LDH and CK-MB were significantly decreased by PC (Fig. 2b, HCHFD + PC *vs.* HCHFD, *p* < 0.05), and a decreasing trend in the ALT level was observed in the HCHFD + PC group (Fig. 2b). These results indicated the hepatoprotective and cardioprotective effects of PC in hypercholesterolemic rats instead of toxicity. BUN and CREA were not changed by PC administration, indicating that PC has no obvious nephrotoxicity (Fig. 2b).

H&E staining results of the liver tissues from the ND group indicated that the hepatic lobule structure was arranged normally, and the hepatic cord was clearly visible, with little fat in the cytoplasm of the hepatocytes. In the HCHFD group, the division of the hepatic lobule was disordered, and the hepatic cells contained many lipid droplets, indicating serious steatosis in the hepatic cells. In the HCHFD + PC group, the hepatic lobule structure was relatively clear, and the number of lipid droplets was reduced in the cytoplasm, indicating that the hepatic pathology was substantially improved by PC treatment (representative pictures are shown in Fig. 2c, and more independent sections are shown in Fig. S1a).

Furthermore, the Oil Red O staining results demonstrated that the number of lipid droplets was significantly increased in the HCHFD group compared to the ND group but significantly decreased in the HCHFD + PC group (Fig. 2c and Fig. S1b). These results indicated that PC relieved hepatic cell vacuolation, reduced lipid droplets in the cytoplasm and effectively protected the liver from steatosis.

Overall, PC effectively ameliorated HCHFD-induced hypercholesterolemia and liver steatosis in rats and showed no toxicity in the duration of the experiment.

### PC altered pathways related to lipid metabolism in vivo

**Fig. 3:**
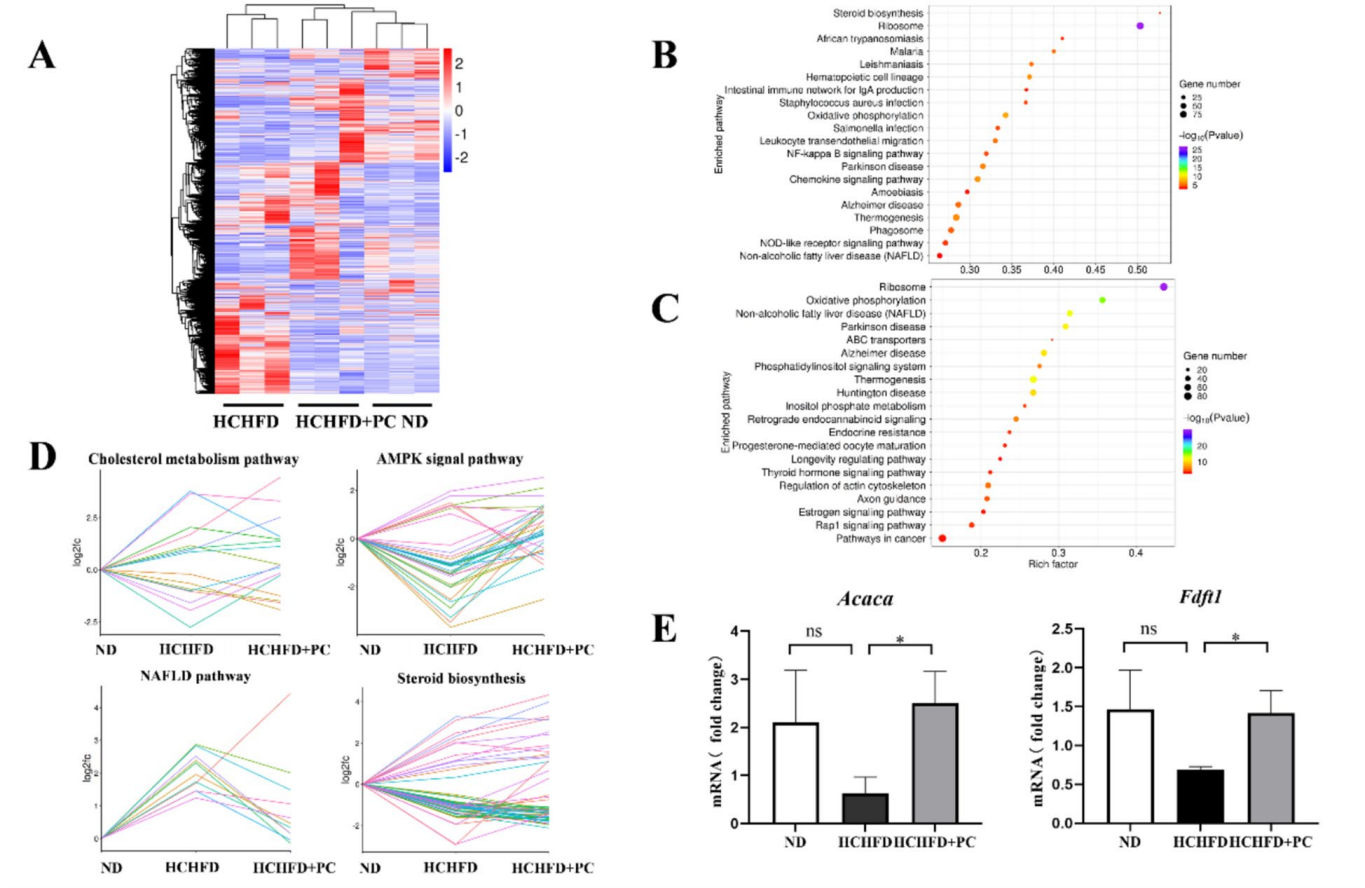
PC altered pathways related to lipid metabolism in vivo. **A** Heatmap of the DEGs in the ND, HCHFD and HCHFD + PC groups. Red: upregulated; blue: downregulated. **B** Bubble chart with the top 20 enriched KEGG pathways for the DEGs of HCHFD *vs.* ND and **C** HCHFD + PC *vs.* HCHFD. The Y-axis label represents the pathway, and the X-axis label represents the rich factor (rich factor = number of DEGs enriched in the pathway/number of all genes in the background gene set). The size and color of the bubble represent the number of DEGs enriched in the pathway and the enrichment significance, respectively. **D** Log_**2**_ (fold change) of the DEGs in four signaling pathways among the three groups (ND, HCHFD, HCHFD + PC). E qRT-PCR validation of *Acaca* and *Fdft1*. *Gapdh* was used as an internal control. Data are shown as the mean ± SD. *p < 0.05, ns: not significant

Transcriptome analysis was performed on liver tissues obtained from the ND, HCHFD, and HCHFD + PC groups (n = 3 from each group) with RNA-seq (The transcriptome dataset is accessible with GEO accession: GSE: 190631). Clustering analysis showed that the replicate samples in each group were clustered together, and the HCHFD + PC group was clustered more closely to the ND group than the HCHFD group (Fig. 3a), indicating that HCHFD altered the expression pattern of many genes, while PC administration reversed these changes.

HCHFD treatment resulted in 6523 DEGs (*vs.* ND group, cutoff: fold change ≥ 2 and *p* value ≤ 0.05): 3943 were upregulated and 2580 were downregulated. A total of 3906 DEGs were identified between the HCHFD and HCHFD + PC groups (1900 upregulated and 2006 downregulated).

The genes dysregulated by HCHFD were significantly enriched in pathways such as steroid biosynthesis and nonalcoholic fatty liver disease (NAFLD, Fig. 3b). This result was not surprising because HCHFD strongly interfered with lipid metabolism in the rat livers. Inspiring, the genes identified in the HCHFD + PC *vs.* HCHFD comparison were enriched in pathways such as NAFLD, phosphatidylinositol signaling system, inositol phosphate metabolism, and ABC transporters (Fig. 3c). These results indicated that PC altered lipid metabolism in the disease model.

To further study the effect of PC on lipid metabolism, we selected some lipid-related pathways, including cholesterol metabolism, the AMPK signaling pathway, NAFLD and the steroid biosynthesis signaling pathway, and assessed the expression of altered genes among the three groups. PC-mediated reversal of the effects of HCHFD was apparent based on the line charts of DEGs in these pathways (Fig. 3d).

*Acaca* (acetyl-CoA carboxylase alpha) from the AMPK signaling pathway and *Fdft1* (farnesyl-diphosphate farnesyltransferase 1) from the cholesterol signaling pathway) were selected and subjected to qRT-PCR validation. We found that the *Acaca* and *Fdft1* mRNA levels in the livers of the HCHFD rats were both reduced compared with those of the control rats, whereas these levels were rescued by PC supplementation (Fig. 3e).

### PC altered pathways related to lipid metabolism in vitro

To further explore the mode of action of PC, we analyzed liver cell lines. Before treating cells with PC, we investigated the effects of PC on cell viability in two liver cell lines: HepG2 and HL-7702 cells. HepG2 and HL-7702 cells were treated with different concentrations of PC for 24 h, and cell viability was detected by CCK8 assays. The growth inhibition curve demonstrated that PC impaired cell viability in a dose-dependent manner (Fig. S2a and S2b). According to the results, PC concentrations of 125 μg/mL and 75 μg/mL were selected for the HepG2 and HL-7702 cell lines, respectively, in all subsequent experiments. The dosages were nearly half of the IC30 values of PC in the HepG2 (210 μg/mL) and HL-7702 (112 μg/mL) cells. At these dosages, cell viability was mildly affected, and serious cytotoxicity was not observed.

**Fig. 4:**
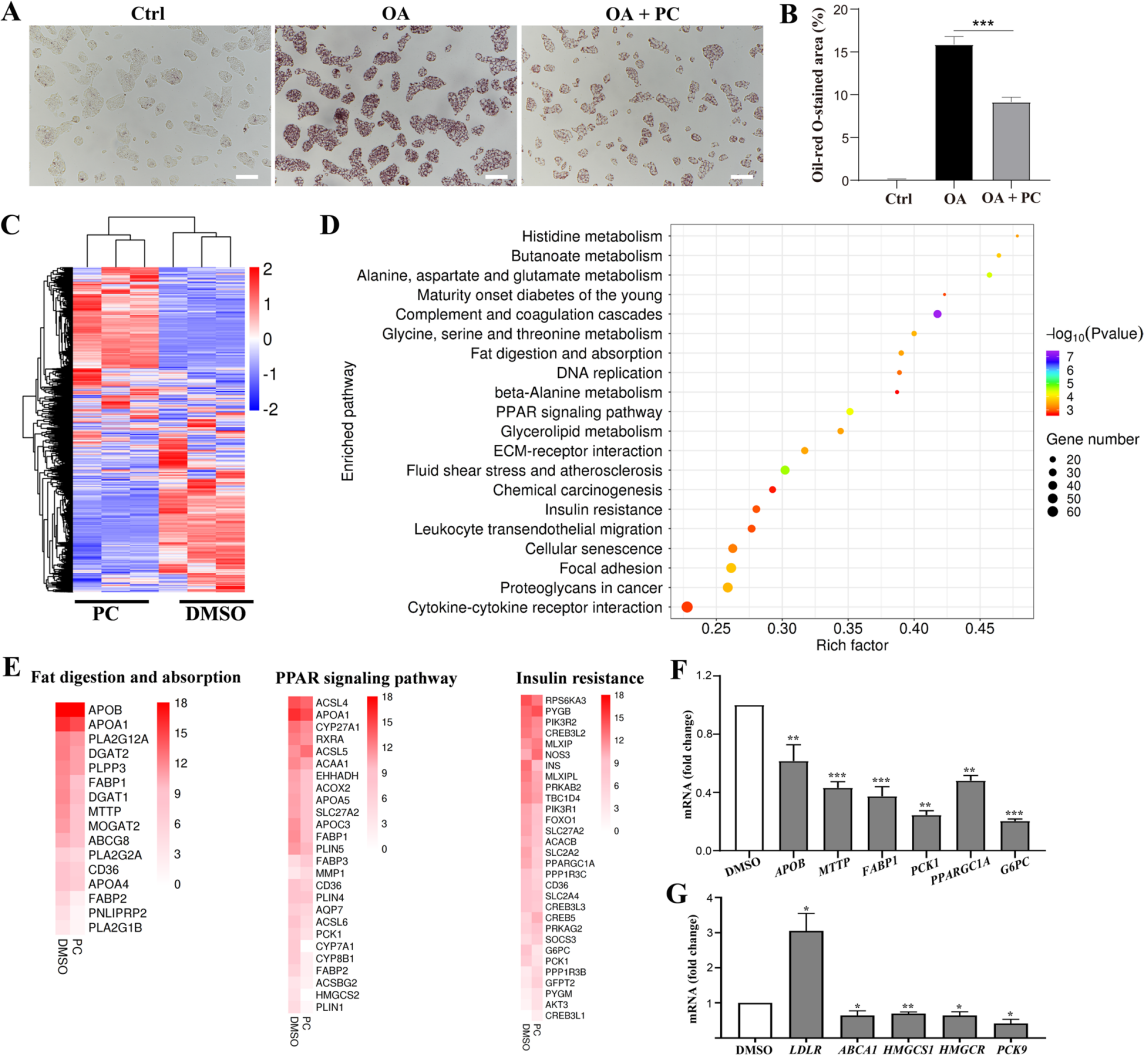
PC altered pathways related to lipid metabolism in vitro. **A** Representative images showing oil-red O stained HepG2 cells under indicated treatments. Scale bar, 100 μm. **B** Statistical analysis of oil-red O-stained area in A (n = 3). **C** Heatmap of the DEGs of PC-treated samples compared to the DMSO-treated samples. Red: upregulated; blue: downregulated. **D** KEGG pathway analysis of two groups (PC *vs.* DMSO) of the DEGs. The bubble chart shows the enrichment of the DEGs in the top 20 signaling pathways. The Y-axis label represents the pathway, and the X-axis label represents the rich factor (rich factor = number of differentially expressed genes enriched in the pathway/number of all genes in background gene set). The size and color of the bubble represent the number of DEGs enriched in the pathway and the enrichment significance, respectively. **E** Heatmap of the DEGs implicated in fat digestion and absorption, the PPAR signaling pathway and the insulin resistance signaling pathway. The color of each hot spot in the heatmap corresponds to the log_2_ (average expression value) of each gene, from white (low expression level) to red (high expression level). **F**, **G** qRT-PCR analysis of some key genes related to the fat digestion and absorption, PPAR, insulin resistance pathways (**F**) and cholesterol metabolism (**G**) after PC (125 μg/mL) treatment in HepG2 cells (n = 3). Data are shown as the mean ± SD. *p < 0.05; **p < 0.01;***p < 0.001. PC *vs.* DMSO or as indicated

Next, the effect of PC to mitigate hyperlipidemia was confirmed in vitro. Oleic acid (OA) was exploited to induce a hyperlipidemia status in HepG2 cells. OA treatment led to intracellular lipid accumulation, as indicated by oil-red O staining (Fig. 4a). In contrast, the addition of PC dramatically decreased the intracellular lipid level, which was evidenced by significantly decreased oil-red O staining area (Fig. 4a, b). These results further confirmed that PC exerts anti-hyperlipidemia activity.

To identify key targets underlying the cholesterol-lowering effect of PC, we incubated HepG2 cells with 125 μg/mL PC for 24 h and subjected them to RNA-seq (GEO accession:GSE:190631). Clustering analysis showed that the PC group and the DMSO group were clearly separated from each other (Fig. 4c). A total of 3363 upregulated genes and 3932 downregulated genes were identified compared with the DMSO control group (cutoff: fold change ≥ 2 and *p* value ≤ 0.05).

Pathway analysis revealed that the genes dysregulated by PC in HepG2 cells were enriched in pathways such as alanine, aspartate and glutamate metabolism; glycine, serine and threonine metabolism; fat digestion and absorption; the glucose-lipid metabolism signaling pathway; the PPAR signaling pathway; and the insulin resistance signaling pathway (Fig. 4d). These results indicated that PC orchestrated pathways involved in glucose and lipid metabolism. A heatmap of DEGs implicated in three important pathways, *i.e.*, fat digestion and absorption metabolism, the PPAR signaling pathway, and the insulin resistance signaling pathway, also suggested the regulatory effect of PC (Fig. 4e). Representative genes were selected for qRT-PCR validation, including *APOB*, *MTTP* (from the fat digestion and absorption signaling pathway), *FABP1*, *PCK1* (from the PPAR signaling pathway) and *PPARGC1A*, *G6PC* (from the insulin resistance signaling pathway). As shown in Fig. 4f, PC treatment downregulated the mRNA levels of all these genes, which is consistent with the results of RNA-seq.

According to the in vivo study, PC significantly reduced serum TC and LDL-c levels in hyperlipidemic animals, several key genes from the cholesterol metabolism signaling pathway were especially selected for qRT-PCR validation. We found that PC downregulated the expression of ATP binding cassette subfamily A member 1 (*ABCA1*), 3-hydroxy-3-methylglutaryl-CoAreductase (*HMGCR*), 3-hydroxy-3-methylglutaryl-CoA synthase 1 (*HMGCS1*), and proprotein convertase subtilisin/kexin type 9 (*PCSK9*) while upregulating *LDLR* (Fig. 4g).

### PC promoted LDL uptake through the upregulation of LDLR

**Fig. 5:**
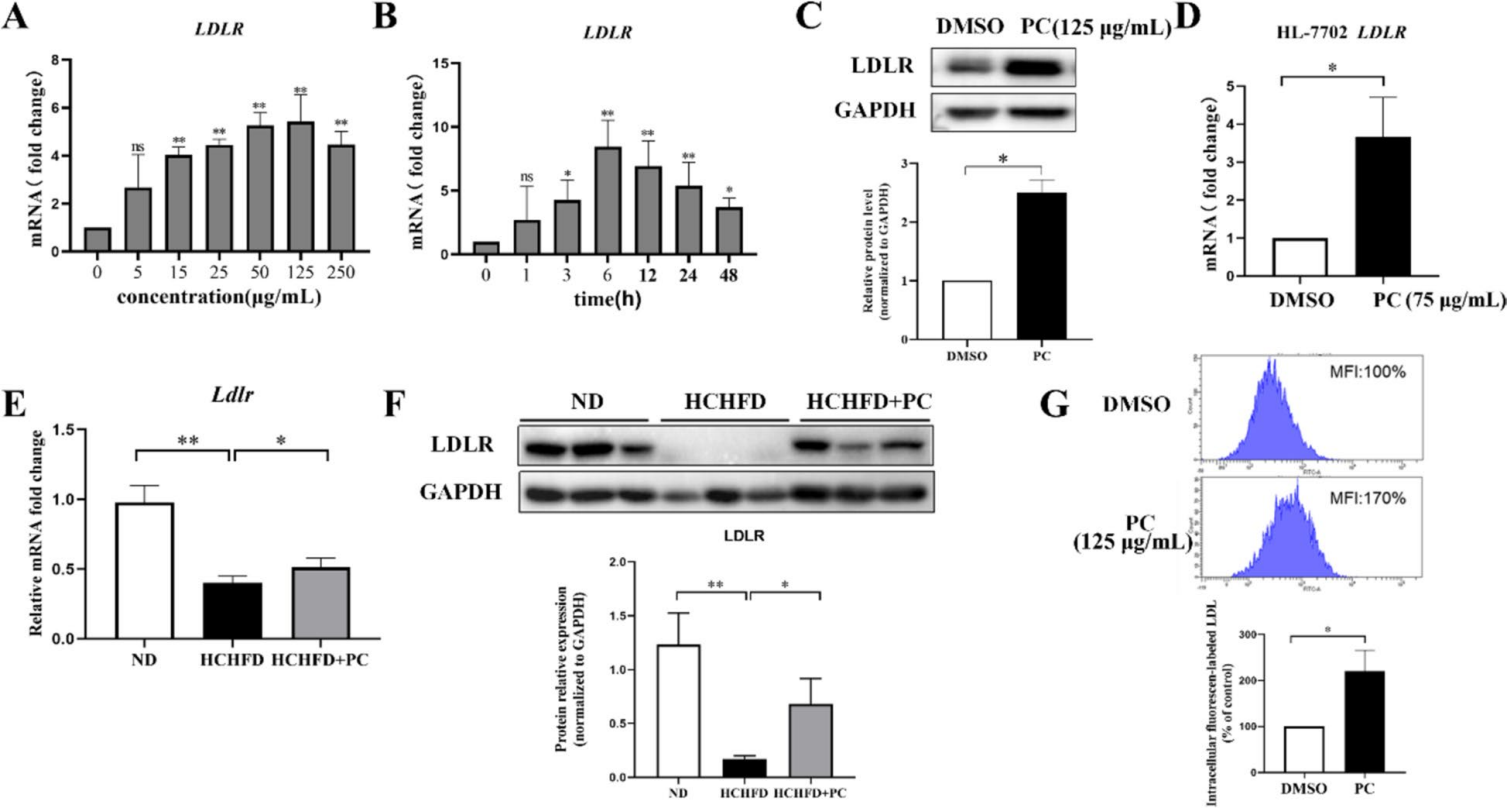
PC promoted LDL uptake through the upregulation of LDLR. **A** Dose–response effect of PC on LDLR expression in HepG2 cells (n = 3). **B** Time course of PC on LDLR expression in HepG2 cells (n = 4). **C** Representative Western blot analysis of LDLR and quantification of three independent Western blot analyses of HepG2 cells (n = 3). **D** Effect of PC on the *LDLR* mRNA level in HL-7702 cells (n = 3). **E** qRT-PCR analysis of the liver *Ldlr* gene in the rats from the three groups (n = 4). **F** Western blot analysis of the LDLR protein level in liver tissues and quantification of Western blots of three independent (n = 3) rat liver samples. G Representative result of LDL uptake and quantification result of three independent experiments (n = 3) for LDL uptake capacity of HepG2 cells. MFI: mean fluorescence intensity. Data are shown as the mean ± SD. For **A**, **B** statistical analyses were conducted using one-way analysis of variance (ANOVA) followed by Dunnett significant difference test. *p < 0.05; **p < 0.01. PC *vs.* DMSO or as indicated. The concentrations of PC for HepG2 and HL-7702 cell treatment were 125 μg/mL and 75 μg/mL, respectively

Since LDLR is a key player in cholesterol homeostasis and mediates the clearance of LDL-c in blood, we aimed to explore the mechanism by which PC regulates its expression. HepG2 cells were cultured with increasing concentrations of PC, and the qRT-PCR results showed that PC increased the *LDLR* mRNA level in a dose-dependent manner in vitro and that the maximal induction of *LDLR* mRNA was achieved at 125 µg/mL (Fig. 5a).

The time course of the PC-induced increase in *LDLR* mRNA was also determined by qRT-PCR. The increase in *LDLR* mRNA was highest after 6 h of PC treatment, approximately 8.5-fold, and was maintained at 3.7-fold after 48 h (Fig. 5b). The PC-mediated induction of LDLR expression was further confirmed by Western blots of HepG2 cells (Fig. 5c and Fig. S3) after 125 µg/mL treatment for 24 h. Another hepatic cell line, HL-7702, was also used to test the expression of *LDLR* under PC treatment. The qRT-PCR results showed that PC at 75 µg/mL increased the *LDLR* mRNA in HL-7702 cells by 3.6-fold. (Fig. 5d).

Consistent with the in vitro results, the *LDLR* mRNA level was increased in the rat livers in the HCHFD disease model after PC administration (Fig. 5e). The protein level of LDLR which was downregulated in the hyperlipidemic rats was also rescued by PC treatment (Fig. 5f and Fig. S4).

Cholesterol often exists in the form of lipoproteins in the blood, and LDL is the main carrier of endogenous cholesterol transport. Approximately 75% of LDL-c in circulation enters intracellular metabolism and mainly relies on the LDLR located in the hepatocyte membrane through receptor-mediated endocytosis [3, 40]. To investigate whether the upregulation of LDLR by PC treatment led to an increase in the LDL absorption capacity, we examined the uptake of BODIPYTM FL-labeled LDL in HepG2 cells with or without PC incubation. The results showed that the fluorescence intensity in the PC-treated cells was significantly higher than that in the DMSO-treated cells (Fig. 5g). PC treatment effectively enhanced the uptake of LDL, indicating that the upregulation of LDLR by PC treatment did achieve an increase in the LDL absorption capacity of cells.

### PC upregulated LDLR expression in an ERK-dependent manner

**Fig. 6:**
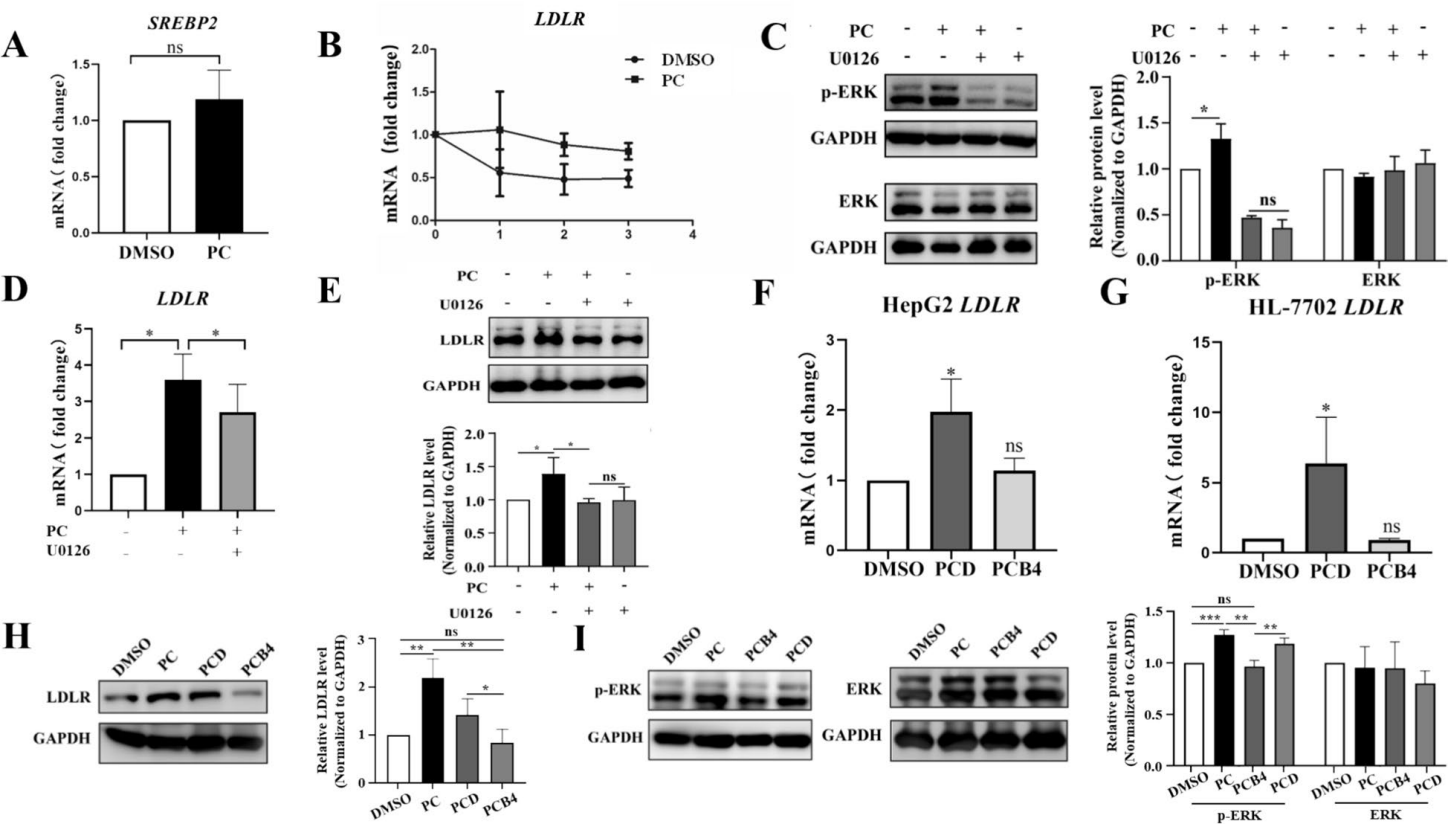
PC and PCD upregulates LDLR expression in an ERK dependent manner. **A** qRT-PCR analysis of *SREBP2* under PC (125 μg/mL) treatment in HepG2 cells (n = 7). **B** HepG2 cells were treated with PC or DMSO for 6 h followed by actinomycin **D** (5 μg/mL) treatment for 1 h, 2 h or 3 h. *LDLR* mRNA was determined by qRT-PCR (n = 3). **C** Representative Western blot analysis of t-ERK and p-ERK and quantification of three independent Western blot analyses (n = 3). **D** The effect of the ERK inhibitor U0126 on PC-induced *LDLR* upregulation at the mRNA level (n = 3). **E** The effect of the ERK inhibitor U0126 on the PC-induced LDLR protein levels and ERK phosphorylation (n = 3). **F** The effect of PCD and PCB4 on the *LDLR* mRNA levels in HepG2 cells (n = 3). **G** The effect of PCD and PCB4 on the *LDLR* mRNA levels in HL-7702 cells (n = 3). **H** Representative result of the Western blot analysis of LDLR protein and quantification of three independent Western blot analyses in HepG2 cells (n = 3). **I** Representative Western blot analysis of t-ERK and p-ERK and quantification of four independent Western blot analyses of HepG2 cells (n = 4). Each result represents the mean ± SD. A-E: *p < 0.05; **p < 0.01. F-I: *p < 0.05. *vs*. DMSO as indicated

LDLR expression is tightly regulated through multiple mechanisms. First, we examined whether PC induced LDLR through the activation of SREBP2. SREBP2 regulates genes involved in cholesterol synthesis by binding to the sterol response element (SRE) and can be modulated by drugs such as statins. We found that PC did not affect the *SREBP2* mRNA level (Fig. 6a). Downstream targets of SREBP2, such as *HMGCR* and *HMGCS1*, were not upregulated by PC either. In contrast, we found that *HMGCR* and *HMGCS1* were downregulated by PC. This pattern was similar to that of *C. chinensis* and its bioactive component berberine, which upregulates the *LDLR* mRNA level and downregulates MVA pathway genes based on our previous study [5]. Berberine has been reported to upregulate *LDLR* mRNA expression at the post-transcriptional level by stabilizing its 3′UTR in an ERK-dependent manner [12]. This finding inspired us to hypothesize that PC functions in a similar way.

Based on the mRNA decay assay following actinomycin D treatment, we found that PC dampened the decay of *LDLR* mRNA compared to DMSO (Fig. 6b). Then, we examined the effect of PC on the phosphorylation of ERK in HepG2 cells by Western blots. As shown in Fig. 6c and Fig. S5, the phosphorylation of ERK was significantly increased by PC without affecting the amount of total ERK. Addition of U0126, an ERK inhibitor, reversed the phosphorylated ERK induced by PC. Besides, U0126 abrogated the phosphorylation of ERK to a similar extent both in the presence and absence of PC (Fig. S5).

To explore whether there is a correlation between the upregulation of LDLR and the activation of the ERK signaling pathway by PC treatment, we added U0126. As expected, we found that the upregulation of LDLR expression by PC was abolished by U0126 by both qRT-PCR (Fig. 6d) and Western blots (Fig. 6e and Fig. S6a, b). In addition, the LDLR level in cells treated with PC plus U0126 was similar to those treated with U0126 only (Fig. 6e and Fig. S6a, b). To further confirm that PC promotes LDL uptake via elevating ERK activity-dependent LDLR regulation, cells exposed to PC with or without U0126 were subjected to Dil-LDL uptake assessment. As displayed in Fig. S6c and 6d, PC elevated intracellular MFI compared to the DMSO group, which was reversed by further introduction of U0126, indicating that ERK activation is critical for LDLR-mediated LDL uptake. These results indicated that the activation of the ERK pathway was indispensable for the effect of PC to upregulate LDLR level for enhanced LDL internalization.

Next, a high cholesterol environment was established with cholesterol and its metabolite 25-hydroxycholesterol to further confirm the mechanism [34]. As expected, the level of p-ERK significantly decreased in the high cholesterol environment (the Model group) compared to the control group, which was reversed by further treatment with PC (the Model + PC group) (Fig. S7). In the meanwhile, the amount of total ERK was almost the same among each group (Fig. S7). Moreover, the inhibition of LDLR expression in the Model group was reversed by the addition of PC (Fig. S8), while the presence of U0126 abrogated the ability of PC to upregulate LDLR mRNA level (the Model + PC + U0126) (Fig. S8). These results indicate that PC could upregulate LDLR expression in an ERK-dependent manner in a high cholesterol environment.

### PCD partially represented the effect of PC on LDLR expression

Previous studies demonstrated that the major bioactive constituents of PC are triterpenoid saponins. Among the saponins, PCD and PCB4 are two of the major constituents in PC [41]. In this study, we assessed the functional roles of PCD and PCB4 in both HepG2 and HL-7702 cells.

The growth inhibition curves are shown in Fig. S9. On the basis of these data, the IC50 values of PCD and PCB4 were 3.5 μg/mL and 2555 μg/mL in HepG2 cells, respectively. In HL-7702 cells, the IC50 of PCD was 2.8 μg/mL, while the IC50 of PCB4 was far beyond the dose range we set. Hence, we used 2.5 μg/mL PCD and 200 μg/mL PCB4 in all subsequent experiments in both cell lines.

To investigate whether PCD and PCB4 were the major active ingredients of PC that regulate LDLR, we tested the effects of PCD and PCB4 on LDLR expression. We found that PCD increased the *LDLR* mRNA levels in both HepG2 and HL-7702 cells; however, PCB4 showed no obvious effect (Fig. 6f and 6g). The protein level of LDLR was determined by Western blots of HepG2 cells, and we found that PCD increased LDLR protein to a less extent than PC extract, while PCB4 showed no effect (Fig. 6h and Fig. S10). In addition, activation of the ERK was observed under PCD but not PCB4 treatment based on Western blots of phosphorylated and total ERK (Fig. 6i and Fig. S10). All the results indicated that PCD is the active ingredient of PC and is partially responsible for the regulatory effect of PC on LDLR expression.

## Conclusions

Traditionally, PC has been regarded as a medicinal herb with antitumor, antioxidant, anti-inflammatory, and anti-endotoxin effects [11]. In the present study, we discovered that PC also possessed lipid-lowering effects for the first time.

Formerly, some scholars used polypharmacology to provide the basis for drug repurposing and identify new functions of some old drugs, which can not only save costs but can also bypass safety issues. Connectivity Map (CMap) is a database that collects microarray-based gene expression profiles from small target molecule-treated human cancer cell lines. L1000 is a high-throughput gene expression signature analysis platform that combines ligation-mediated amplification, optically addressed and barcoded microspheres (beads), and a flow cytometric detection system. Novel drugs were discovered based on these platforms [15, 19, 28]. In the same way, we screened thousands of TCMs based on RASL-seq. We proposed PC as a potential metabolic regulatory agent based on its high similarity with *C. chinensis*. This approach can be extended to other TCMs or chemical research.

From in vitro experiments to in vivo studies, we demonstrated the cholesterol-lowering function of PC. As a key target of PC, LDLR was upregulated by PC in an ERK-dependent manner, similar to the effect of berberine, while distinct from that of statins.

Despite its vital medicinal value, PC is regarded as a toxic TCM [10]. Therefore, further study of its active components is essential for safe administration of this medicine [33]. Based on previous reports, PCD and PCB4 both exhibit anticancer activity. However, we found that they showed differences in regulating LDLR. PCD but not PCB4 upregulated LDLR expression in an ERK-dependent manner. The chemical structures of PCD and PCB4 were different (Fig. S11) and determination of how the structure of PCD leads to the pharmaceutical effects is important.

In summary, we revealed the function, mechanism, and bioactive components of PC as a cholesterol-lowering agent, providing a promising option for the prevention and treatment of hypercholesterolemia.

## Supplementary Information

Additional file 1.

Additional file 2.

Additional file 3.

## Data Availability

The transcriptome dataset is accessible with GEO accession: GSE:190631. Supplemental materials were available online. Other information during the current study is available from the corresponding author upon reasonable request.
