# Supplementary material for: Pulsatilla chinensis functions as a novel antihyperlipidemic agent by upregulating LDLR in an ERK-dependent manner
Source: Chin Med. 2024 Dec 19;19:172. doi: 10.1186/s13020-024-01044-3 (PMC11657699; doi:10.1186/s13020-024-01044-3)
Supplement: Supplementary file 1 — Additional file 1. [file 13020_2024_1044_MOESM1_ESM.pptx]

## Slide 1
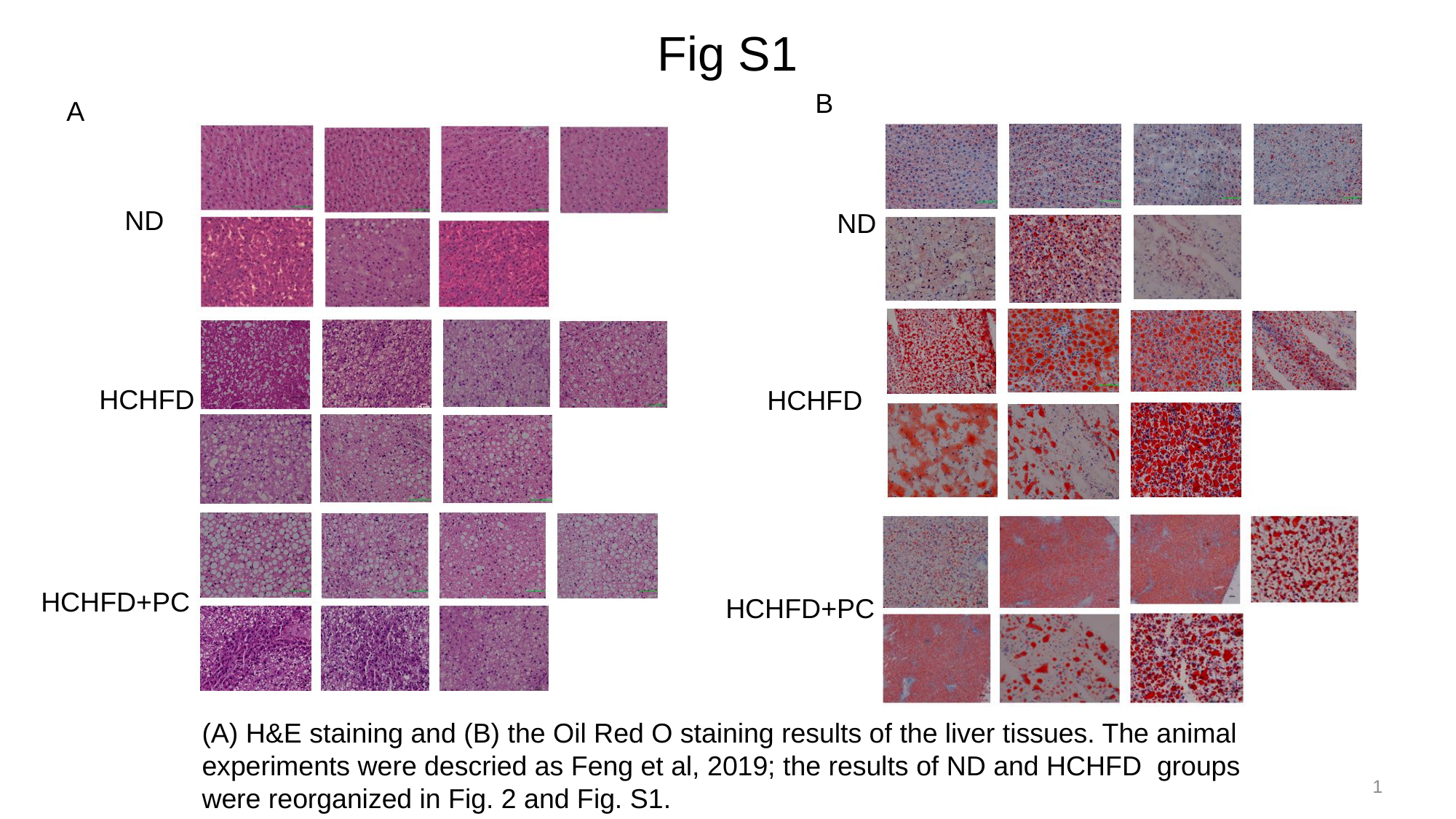

# Fig S1
B
A
ND
ND
HCHFD
HCHFD
HCHFD+PC
HCHFD+PC
(A) H&E staining and (B) the Oil Red O staining results of the liver tissues. The animal experiments were descried as Feng et al, 2019; the results of ND and HCHFD groups were reorganized in Fig. 2 and Fig. S1.
1

## Slide 2
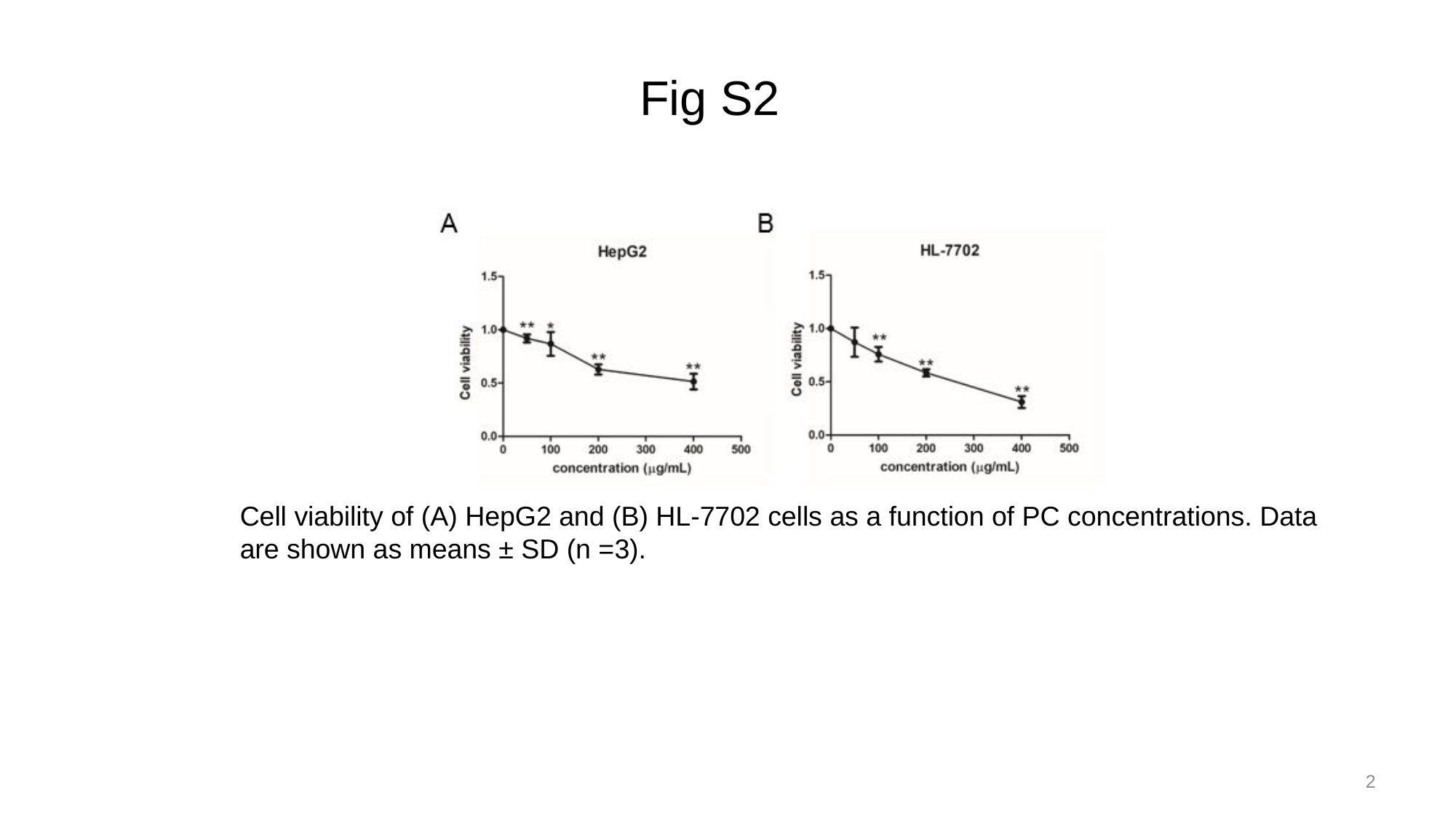

Fig S2
Cell viability of (A) HepG2 and (B) HL-7702 cells as a function of PC concentrations. Data are shown as means ± SD (n =3).
2

## Slide 3
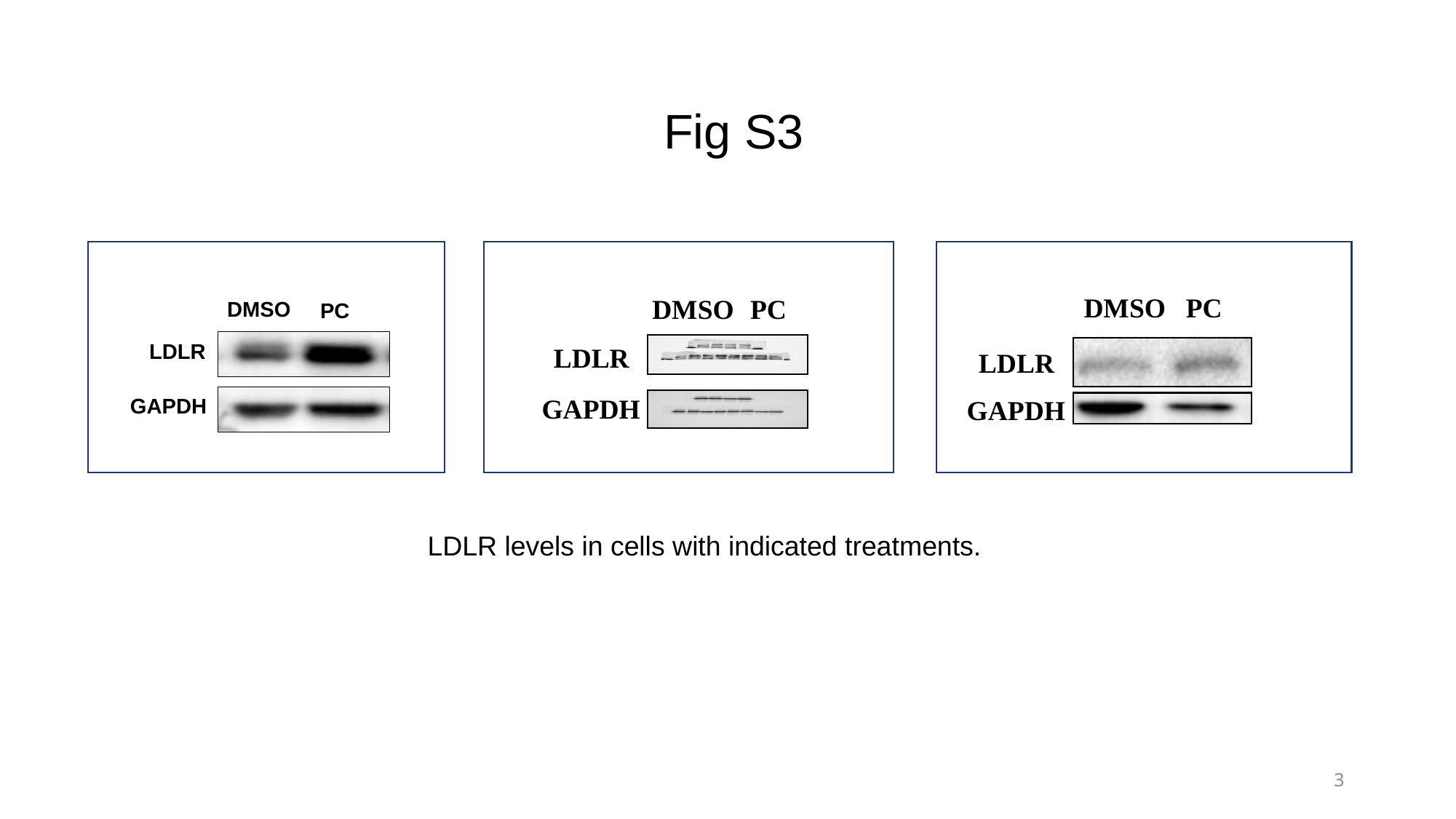

Fig S3
DMSO
PC
DMSO
PC
DMSO
PC
LDLR
GAPDH
LDLR
LDLR
GAPDH
GAPDH
LDLR levels in cells with indicated treatments.
3

## Slide 4
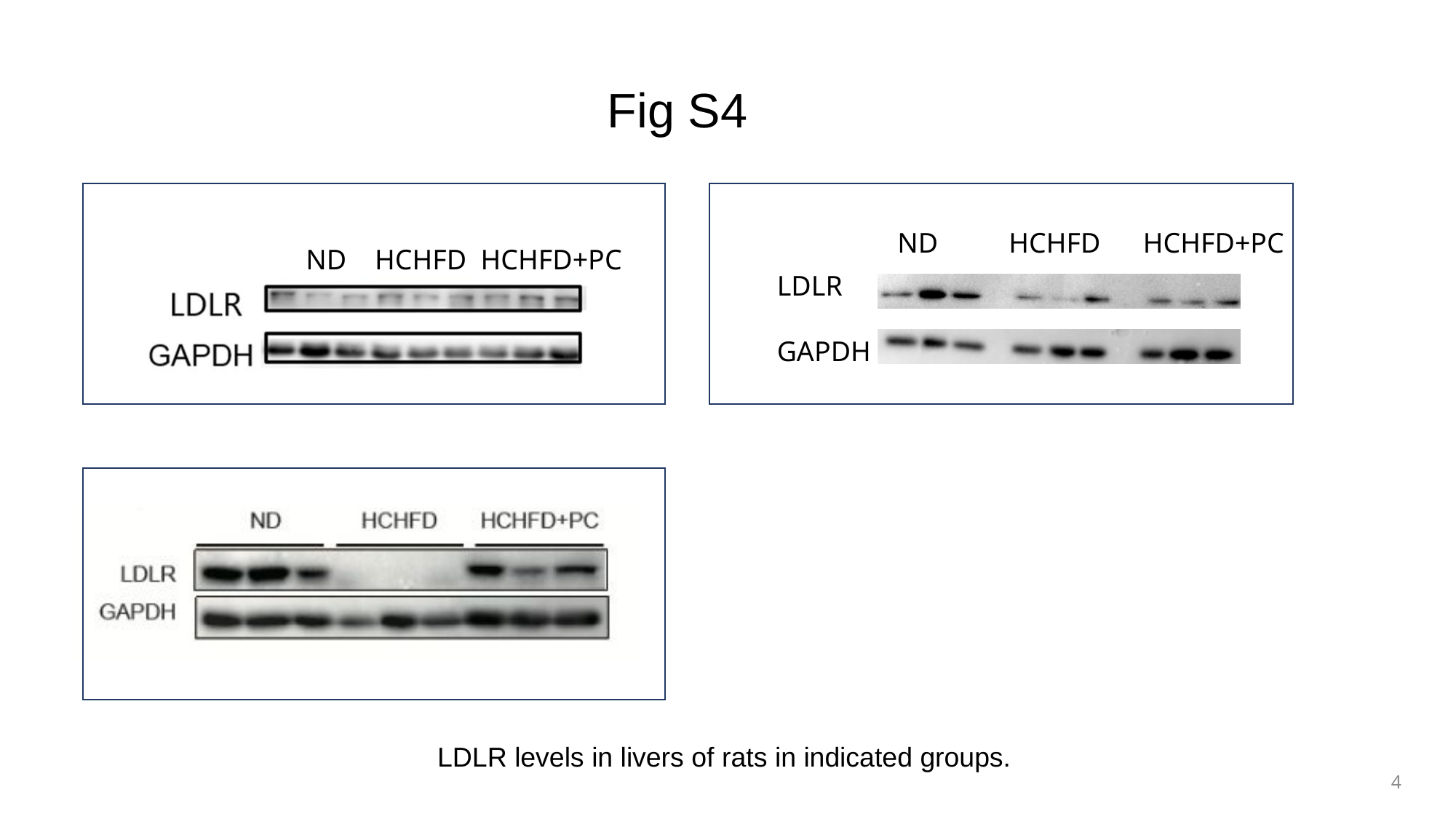

Fig S4
 ND HCHFD HCHFD+PC
 ND HCHFD HCHFD+PC
LDLR
GAPDH
LDLR levels in livers of rats in indicated groups.
4

## Slide 5
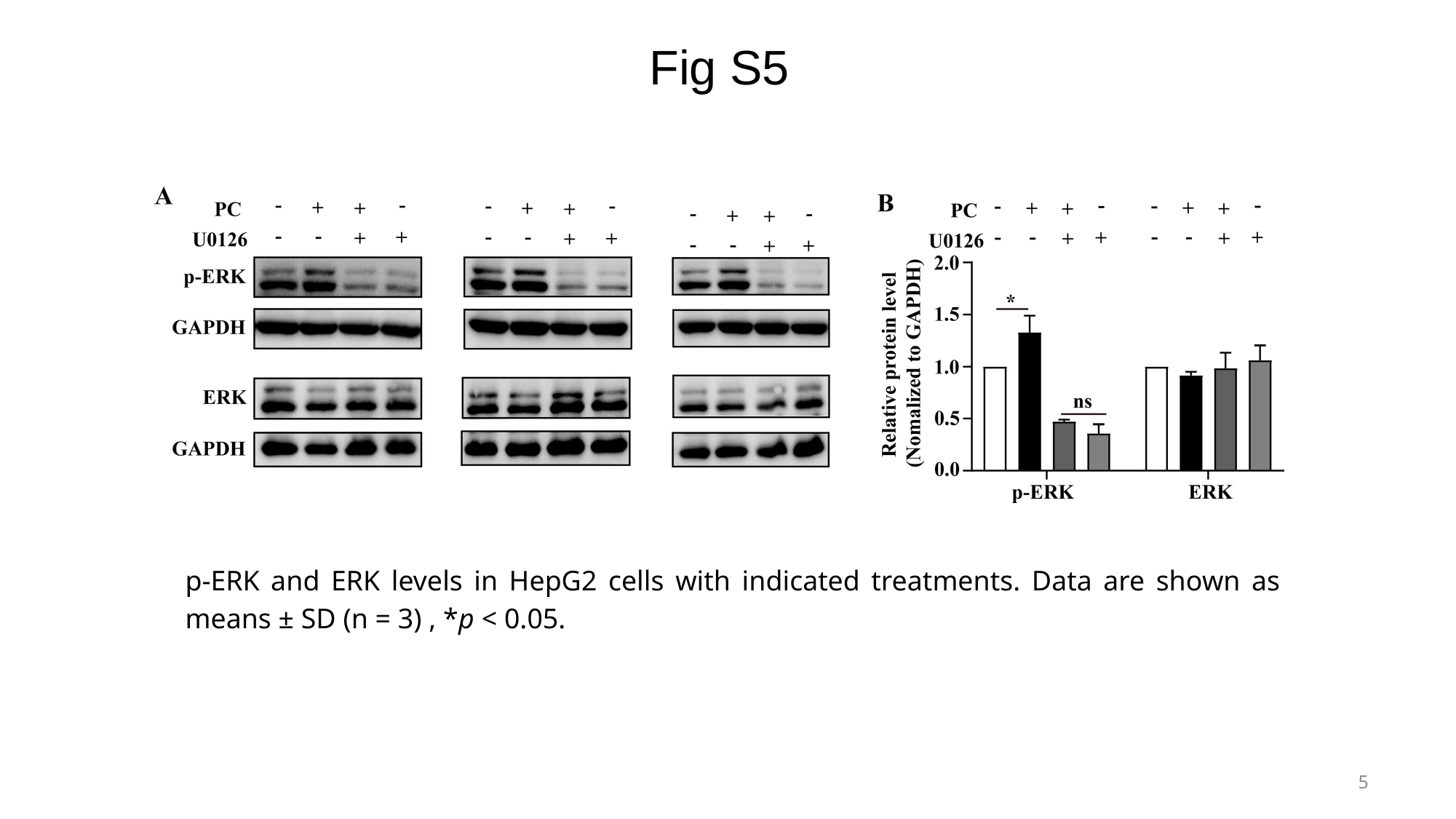

Fig S5
p-ERK and ERK levels in HepG2 cells with indicated treatments. Data are shown as means ± SD (n = 3) , *p < 0.05.
5

## Slide 6
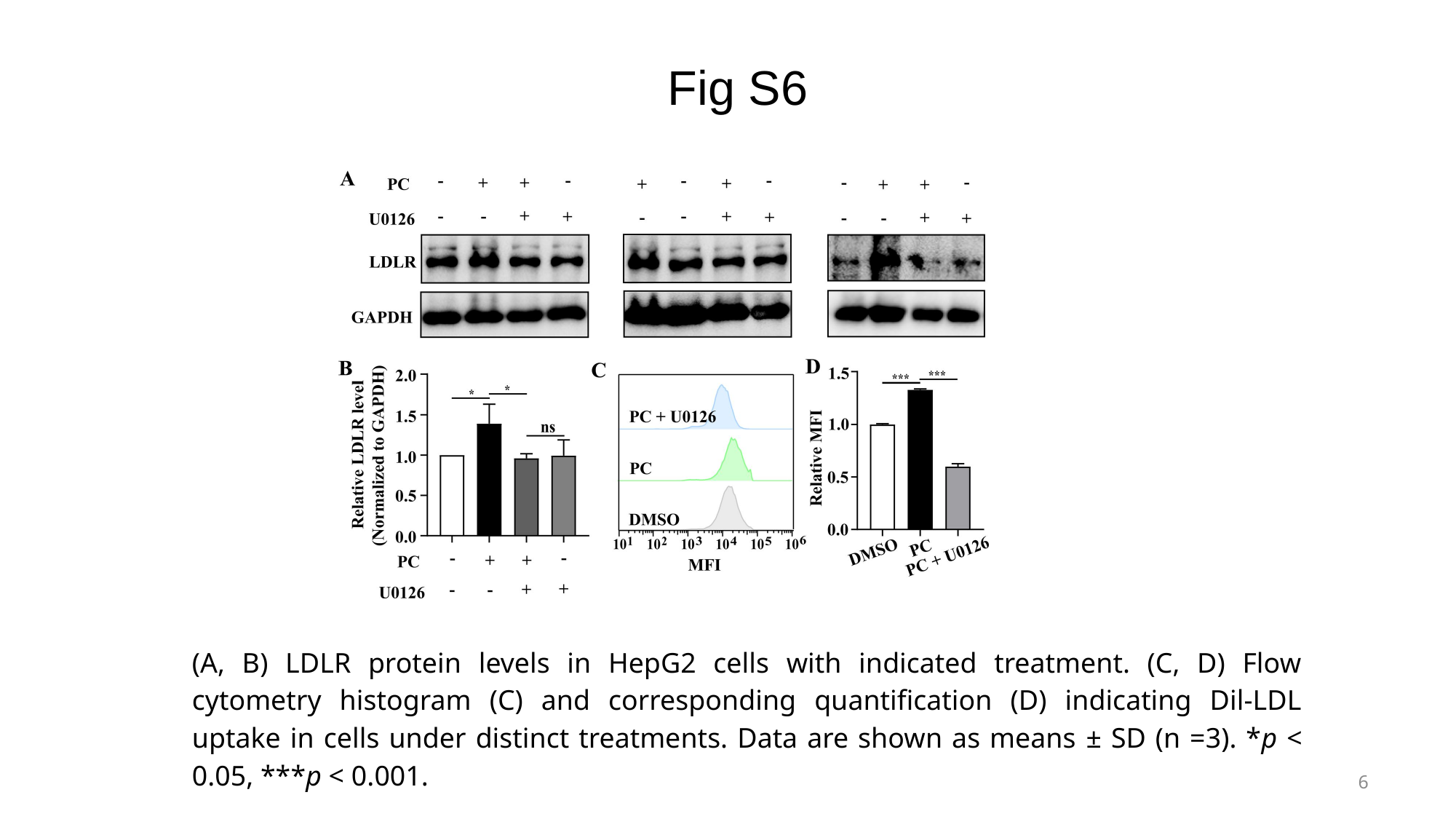

Fig S6
(A, B) LDLR protein levels in HepG2 cells with indicated treatment. (C, D) Flow cytometry histogram (C) and corresponding quantification (D) indicating Dil-LDL uptake in cells under distinct treatments. Data are shown as means ± SD (n =3). *p < 0.05, ***p < 0.001.
6

## Slide 7
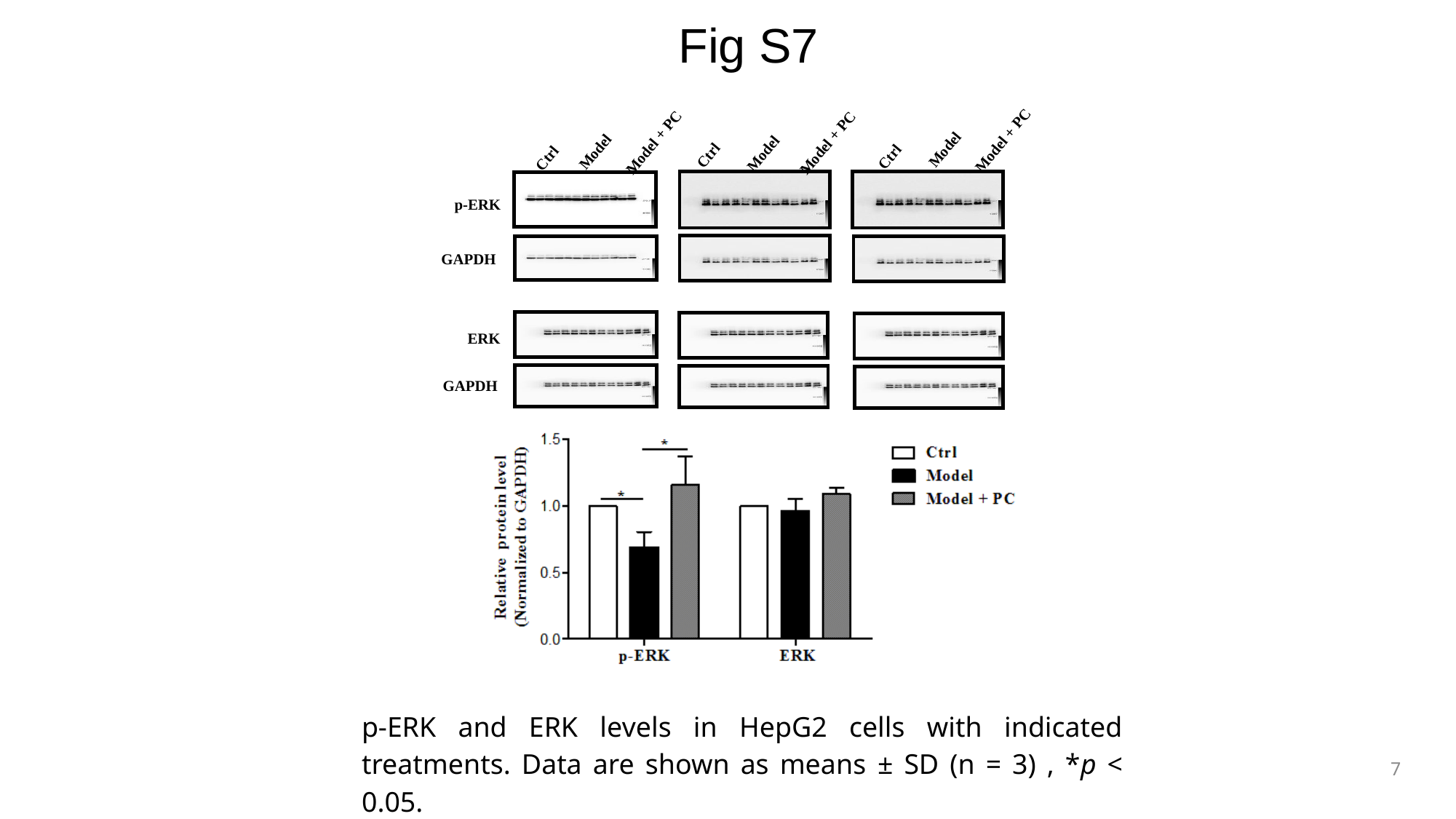

Fig S7
Model + PC
Model + PC
Model + PC
Model
Model
Model
Ctrl
Ctrl
Ctrl
p-ERK
GAPDH
ERK
GAPDH
p-ERK and ERK levels in HepG2 cells with indicated treatments. Data are shown as means ± SD (n = 3) , *p < 0.05.
7

## Slide 8
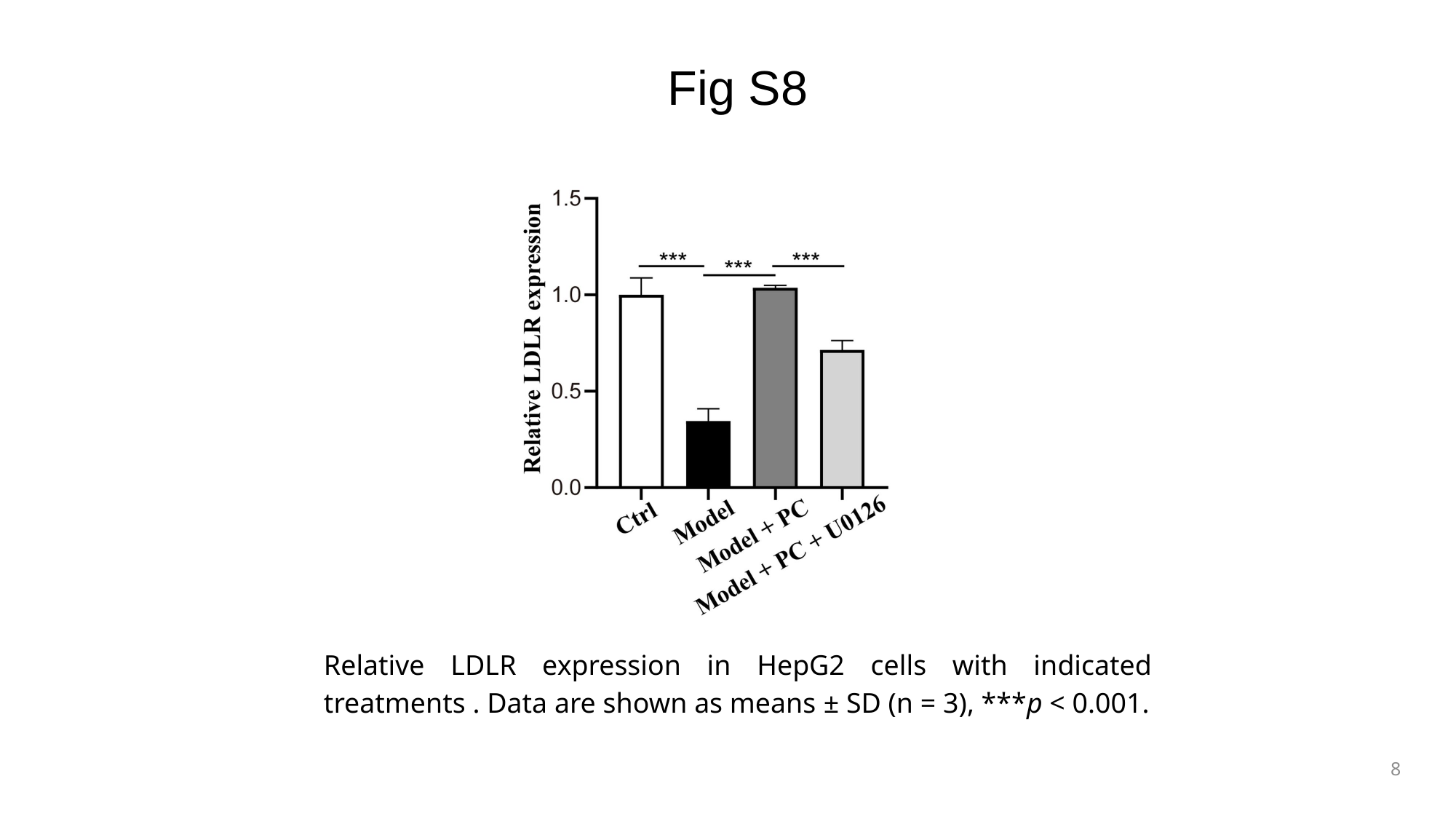

Fig S8
Relative LDLR expression in HepG2 cells with indicated treatments . Data are shown as means ± SD (n = 3), ***p < 0.001.
8

## Slide 9
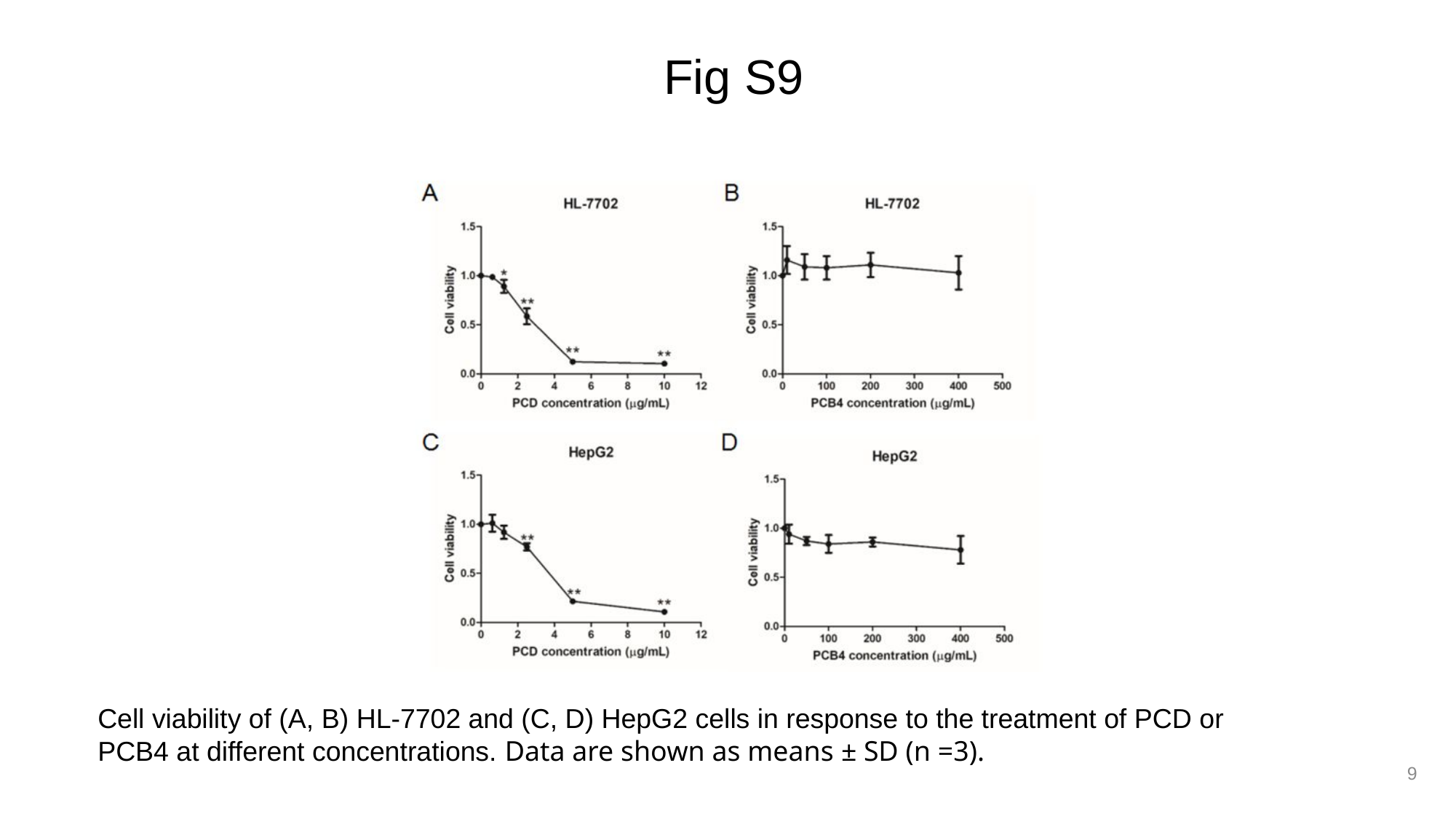

Fig S9
Cell viability of (A, B) HL-7702 and (C, D) HepG2 cells in response to the treatment of PCD or PCB4 at different concentrations. Data are shown as means ± SD (n =3).
9

## Slide 10
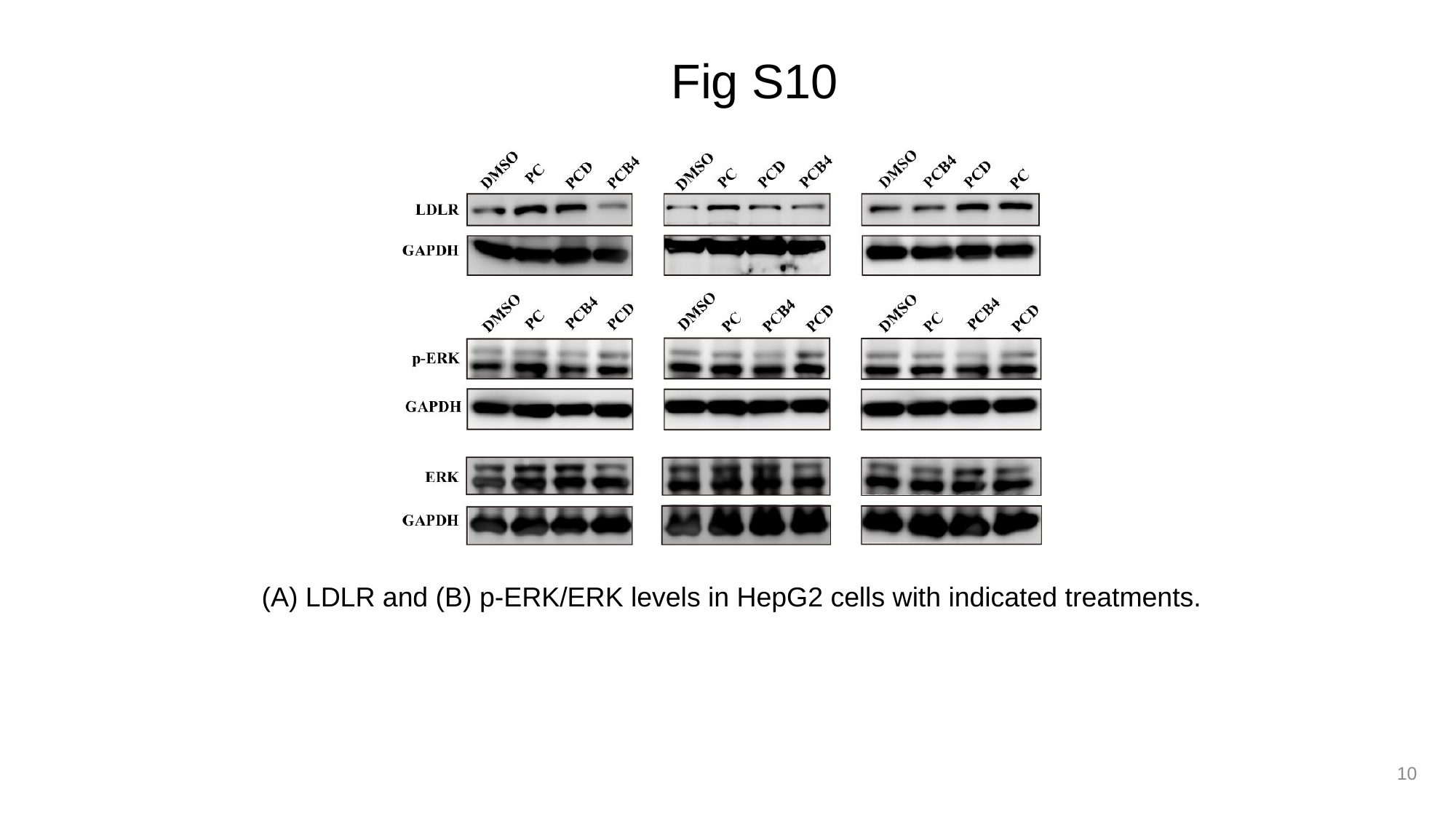

Fig S10
(A) LDLR and (B) p-ERK/ERK levels in HepG2 cells with indicated treatments.
10

## Slide 11
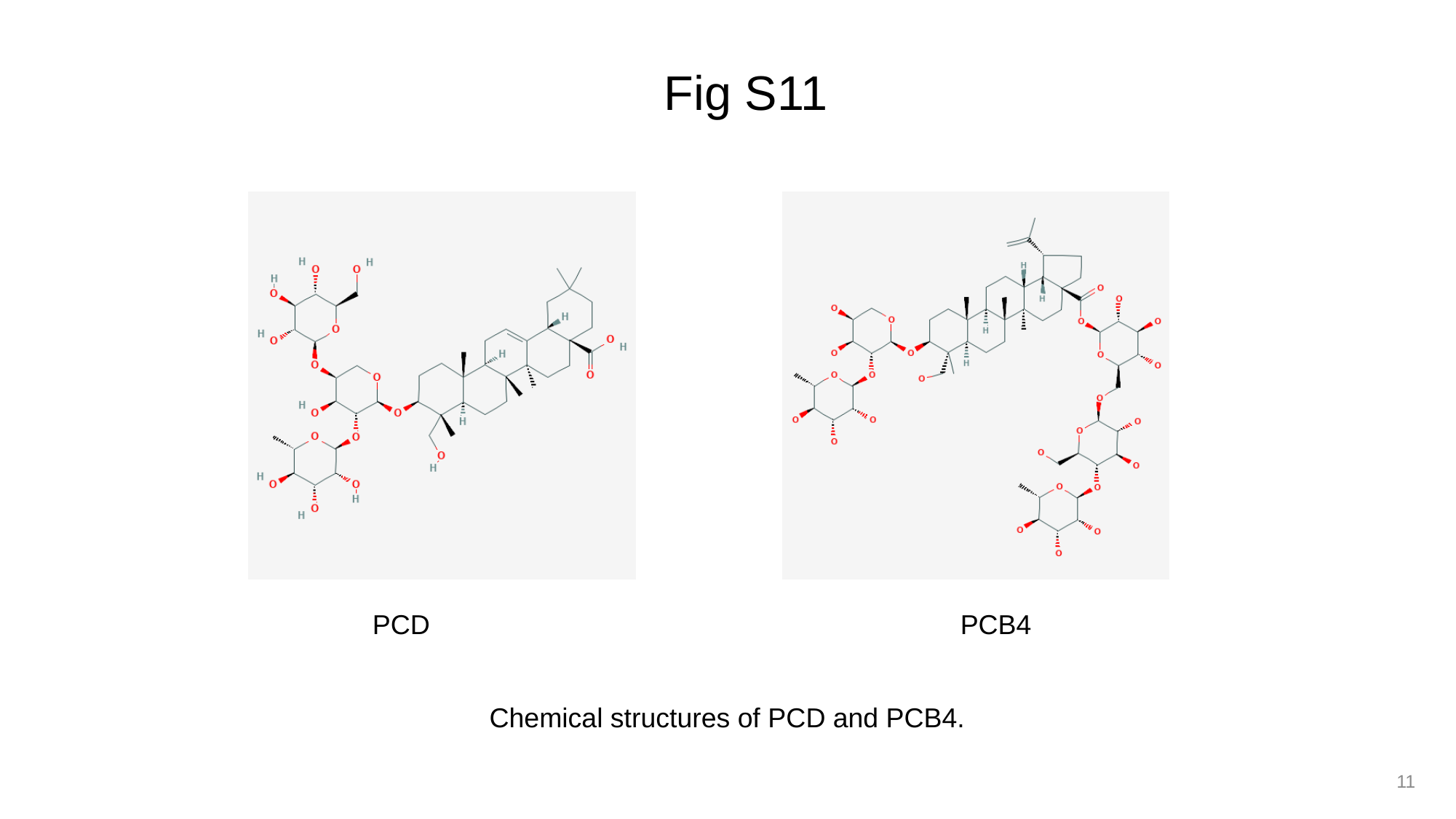

Fig S11
PCD
PCB4
Chemical structures of PCD and PCB4.
11
